# A systematic literature review of the human papillomavirus prevalence in locally and regionally advanced and recurrent/metastatic head and neck cancers through the last decade: The “ALARM” study

**DOI:** 10.1002/cam4.6916

**Published:** 2024-01-21

**Authors:** Sofia Agelaki, Ioannis Boukovinas, Ilias Athanasiadis, Georgios Trimis, Ioannis Dimitriadis, Lazaros Poughias, Edith Morais, Ugne Sabale, Goran Bencina, Charalampos Athanasopoulos

**Affiliations:** ^1^ Laboratory of Translational Oncology, School of Medicine University of Crete Herakleion Greece; ^2^ Department of Medical Oncology University General Hospital of Herakleion Herakleion Greece; ^3^ Bioclinic Thessaloniki Medical Oncology Unit Thessaloniki Greece; ^4^ Department of Medical Oncology, Mitera Hospital Athens Greece; ^5^ MSD, Medical Affairs Athens Greece; ^6^ MSD, Center for Observational and Real‐World Evidence (CORE) Lyon France; ^7^ MSD, Center for Observational and Real‐World Evidence (CORE) Stockholm Sweden; ^8^ MSD, Center for Observational and Real‐World Evidence (CORE) Madrid Spain

**Keywords:** cancer prevention, cancer risk factors, epidemiology and prevention, head and neck cancer, viral oncology

## Abstract

**Aims:**

The aim of this systematic literature review was to provide updated information on human papillomavirus (HPV) prevalence in locally and regionally advanced (LA) and recurrent/metastatic (RM) head and neck cancer (HNC) worldwide.

**Methods:**

Electronic searches were conducted on clinicaltrials.gov, MEDLINE/PubMed, Embase, and ASCO/ESMO journals of congresses for interventional studies (IS; Phase I–III trials) as well as MEDLINE and Embase for non‐interventional studies (NIS) of LA/RM HNC published between January 01, 2010 and December 31, 2020. Criteria for study selection included: availability of HPV prevalence data for LA/RM HNC patients, patient enrollment from January 01, 2010 onward, and oropharyngeal cancer (OPC) included among HNC types. HPV prevalence per study was calculated as proportion of HPV+ over total number of enrolled patients. For overall HPV prevalence across studies, mean of reported HPV prevalence rates across studies and pooled estimate (sum of all HPV+ patients over sum of all patients enrolled) were assessed.

**Results:**

Eighty‐one studies (62 IS; 19 NIS) were included, representing 9607 LA/RM HNC cases, with an overall mean (pooled) HPV prevalence of 32.6% (25.1%). HPV prevalence was 44.7% (44.0%) in LA and 24.3% (18.6%) in RM. Among 2714 LA/RM OPC patients from 52 studies with available data, mean (pooled) value was 55.8% (50.7%). The majority of data were derived from Northern America and Europe, with overall HPV prevalence of 46.0% (42.1%) and 24.7% (25.3%) across studies conducted exclusively in these geographic regions, respectively (Northern Europe: 31.9% [63.1%]). A “p16‐based” assay was the most frequently reported HPV detection methodology (58.0%).

**Conclusion:**

Over the last decade, at least one quarter of LA/RM HNC and half of OPC cases studied in IS and NIS were HPV+. This alarming burden is consistent with a potential implication of HPV in the pathogenesis of at least a subgroup of HNC, underscoring the relevance of HPV testing and prophylaxis to HNC prevention and management.

## 1 INTRODUCTION

Head and neck cancer (HNC) accounted for ~5% of new cancer cases and cancer‐related deaths worldwide in 2020, with an estimated annual burden of 931,931 incident cases and 467,125 deaths.^1^ Most HNCs arise from the squamous epithelium of the oral cavity, oropharynx (OPX), larynx, and hypopharynx, collectively referred to as head and neck squamous cell carcinoma (HNSCC).^2^ HNSCC incidence varies across regions, generally reflecting the diverse epidemiology of risk factors such as consumption of tobacco, alcohol, and areca nut.^2^ Another cause of cancers developed in the head and neck (HN) is human papillomavirus (HPV) infection.^3^, ^4^ Among HNC subtypes, HPV has been most frequently associated with oropharyngeal squamous cell carcinoma (OPSCC)^5^, ^6^, ^7^ and is considered to account to a great extent for the increasing incidence of oropharyngeal cancer (OPC) in several high‐income countries over the past decades.^8^, ^9^, ^10^

Besides its etiological role, HPV is also recognized as a prognostic factor in OPC^11^, ^12^, ^13^, ^14^, ^15^, ^16^ and is utilized in the therapeutic algorithm of OPC patients.^17^, ^18^ This highlights the clinical utility of HPV testing in HNC, which, in the absence of diagnostic tests with regulatory approval for use in HNC, is performed by various methodologies.^19^ Each of the available techniques has specific limitations; thus, a combined approach using multiple protein or nucleic acid‐based methods has been suggested for optimal detection of potentially causative HPV.^19^, ^20^ Testing of p16 is recommended by the European Head and Neck Society/European Society for Medical Oncology/European Society for Radiotherapy and Oncology, the National Comprehensive Cancer Network, the College of American Pathologists, and the American Society of Clinical Oncology guidelines as a surrogate HPV biomarker in OPSCC management.^12^, ^13^, ^21^, ^22^, ^23^ Nevertheless, given than the prognostic impact of HPV in other HNC subsites is unclear,^24^, ^25^, ^26^ routine examination for HPV presence for other HNC types is not warranted.^21^

For patients with limited or early‐stage HNSCC, current treatment modalities are potentially curative, while for recurrent and/or metastatic (RM) HNSCC, treatment is complex, prognosis is poor, and the burden on quality of life and productivity can be substantial.^27^, ^28^ Importantly, more than 50% of HNSCC patients present with locally and regionally advanced (LA) or metastatic HNSCC at diagnosis, while recurrence rates for LA HNSCC are high.^28^, ^29^, ^30^ The proportion of those patients who are HPV‐positive (HPV+) remains unknown, as available data are not only outdated, but also mainly refer to the totality of HNCs, not distinguishing the disease by stage.^5^, ^31^, ^32^, ^33^, ^34^, ^35^, ^36^, ^37^ This information is particularly relevant considering that available HPV prophylactic vaccines have shown preliminary efficacy against HN infections, opening an opportunity for primary prevention of the specific cancers,^38^, ^39^, ^40^, ^41^, ^42^ with this potential being investigated in ongoing Phase III clinical trials.^43^, ^44^

Given the challenges in the management of LA and RM HNC, and the increasing incidence of HPV‐associated HNC, updated information on the HPV prevalence is essential, with possible implications for preventive interventions. This systematic literature review (SLR) primarily aimed to enhance understanding of HPV prevalence in LA and/or RM HNC based on evidence from the last decade (2010 to 2020). Additionally, HPV prevalence in LA and RM OPC, geographic distribution of HPV prevalence, and level of homogeneity between HPV testing methodologies were explored.

## 2 METHODS

This SLR was conducted and outcomes were reported in accordance with PRISMA guidelines (see Data S1). The study protocol was registered with PROSPERO, the international prospective register of systematic reviews (registration number: CRD42021256876) and is publicly available.

### 2.1 Information sources and search strategy

Identification of studies in LA and RM HNC was performed separately for IS (i.e., Phase I–III trials) and NIS. For IS, electronic searches were conducted on Clinicaltrials.gov using the keywords “Head and Neck” in combination with “Local”, “Regional”, “Advanced”, “Recurrent”, or “Metastatic” for Phase 1, 2, and 3 studies starting on or after January 01, 2010 until December 31, 2020. The corresponding National Clinical Trial (NCT) numbers were used to search PubMed and Embase databases as well as ASCO/ESMO journals of congresses for related articles and/or abstracts with available results. For NIS, MEDLINE via PubMed and Embase databases were searched for related publications using Medical subject heading terms and keywords developed for disease (HNC), outcome of interest (HPV), relevant cancer type (OPC) to expand search results, disease stage (local, regional, recurrent, metastatic, and advanced), and study design (epidemiology, real‐life, non‐interventional, and observational). The searches were restricted using embedded filters to publications from the last 10 years (January 01, 2010 to December 31, 2020). They were also restricted to articles published in English language and to studies conducted in “humans,” while congress abstracts and reviews were excluded. The detailed search strategy including search strings and resulting number of hits is provided in Data S2. Electronic searches for both IS and NIS were completed on March 19, 2021.

### 2.2 Study selection

Studies were selected based on prespecified eligibility criteria designed according to the PICOTS (population, intervention, comparisons, outcome, time, and study design) framework. Specifically, studies were selected if patients with RM and/or LA HNC had participated, OPX was included among the HN subsites, and HPV status of cancer was available, even if only the OPC subpopulation had been tested for HPV (population). There were no restrictions as to the intervention and comparator of the study, as long as they were intended for disease treatment and not management of safety events of previous therapies (intervention; comparator). Only studies with available results on HPV prevalence (i.e., prevalence of HPV‐related HNC), and/or on the number of HPV+ HNC patients, allowing the calculation of corresponding prevalence were selected (Outcome). Studies initiating enrollment of participants prior to January 01, 2010 were excluded (time). IS of any design were included as long as there was no prerequisite regarding the proportion of patients per HN subsite that needed to be enrolled and NIS of any design and direction of temporal observation (Study design). Articles published in a language other than English were excluded. Finally, only original, peer‐reviewed articles published in scientific journals were selected, with the exception of abstracts published in ASCO/ESMO congress abstract books for IS which were also included. Non‐original studies such as literature reviews were excluded. For IS for which full manuscripts were pending and corresponding abstracts in ASCO/ESMO congress abstract books were available, selection was based on information included in those abstracts. Study design and results captured in Clinicaltrials.gov were utilized cumulatively with manuscripts and/or abstracts available in ASCO/ESMO journals of congresses for selecting IS.

For study selection, an initial screening of titles/abstracts was performed against each eligibility criterion followed by examination of the full‐text article if a definite decision could not be made. Study review and selection was performed by two reviewers working independently (Athena Georgilis and Maria‐Filothei Lazaridou from Qualitis SA). The decisions of the reviewers were compared and any conflicts were resolved by a third reviewer (Charalampos Athanasopoulos for NIS and Georgios Trimis for IS).

### 2.3 Data extraction and analysis

Data from each of the studies that met the predefined eligibility criteria were extracted and cross‐checked by two independent reviewers with respect to the following variables: study design, country, study period, study population including disease stage, age, HPV status detection methodology, subsites where HPV status was assessed (any included site or only OPX), number of LA and/or RM HNC patients enrolled (“N_HNC_ enrolled”), number of HPV+ LA and/or RM HNC patients (“N_HNC_ HPV+”) and/or HPV prevalence (%) in LA and/or RM HNC as defined by the author, number of HPV− LA and/or RM HNC patients (“N_HNC_ HPV−”) to reflect missing HPV status data, number of LA and/or RM OPC patients enrolled (“N_OPC_ enrolled”), number of HPV+ LA and/or RM OPC patients (“N_OPC_ HPV+”) and/or HPV prevalence (%) in LA and/or RM OPC as defined by the author.

The primary outcome of HPV prevalence in LA and RM HNC was calculated as the proportion (%) of “N_HNC_ HPV+” over “N_HNC_ enrolled.” Similarly, OPC fraction among LA and/or RM HNC patients was calculated as the proportion (%) of “N_OPC_ enrolled” over “N_HNC_ enrolled,” as available. For the secondary outcome of HPV prevalence in LA and/or RM OPC, HPV prevalence among selected HNC studies was calculated as the proportion (%) of “N_OPC_ HPV+” over “N_OPC_ enrolled,” as available. Hence, the estimated prevalence of HPV in HNC and OPC represented the minimum number of HPV+ patients in the pool of HNC or OPC patients enrolled in each study, respectively, as patients with no available data on HPV status were also included in the denominators (“N_HNC_ enrolled” and “N_OPC_ enrolled”).

Data extracted from selected studies was organized in summary tables and figures using standard Microsoft Excel® functions and descriptively analyzed. No inferential statistical analysis was conducted. Studies were categorized by design in the subgroups of IS or NIS and by HNC disease stage as either LA, RM, or Other, the latter of which included LA and/or RM stage as defined by the author with no further specification or both LA and RM. Studies were also grouped based on geographic region as defined by the International Agency for Research on Cancer.^45^ In estimating the prevalence of HPV or OPC fraction across HNC studies overall and per the above‐described subgroups, mean and median proportion (%) of HPV+ or OPC patients across studies in each subgroup were calculated. HPV prevalence and OPC fraction overall and per subgroup were also estimated as pooled prevalence, that is, as proportion (%) of the sum of “N_HNC_ or _OPC_ HPV+” or “N_OPC_ enrolled” across studies, respectively, over the sum of “N_HNC_ or _OPC_ enrolled” across studies.

### 2.4 Risk of bias

Taking into account the narrative nature of this SLR and that prevalence of HPV pertains to a baseline patient characteristic, study outcomes are not expected to be affected by the design, conduct, or the statistical power in the results of each included study. To reduce bias with respect to generalizability of HPV prevalence outcomes, during the study selection process, studies with a prespecified patient eligibility criterion regarding HPV status (e.g., HPV+ patients only) or associated with HPV status (e.g., OPC patients only) were excluded. No restrictions were applied with respect to HPV detection methodologies as distribution of different methodologies was an exploratory outcome of interest. Last, the effect of sample size on the primary outcome was examined by visual inspection of the distribution of studies around the overall prevalence of HPV (mean, median, pooled) in a plot of sample size (“N_HNC_ enrolled”) against HPV prevalence.

## 3 RESULTS

### 3.1 Literature search results and characteristics of included studies

**FIGURE 1:**
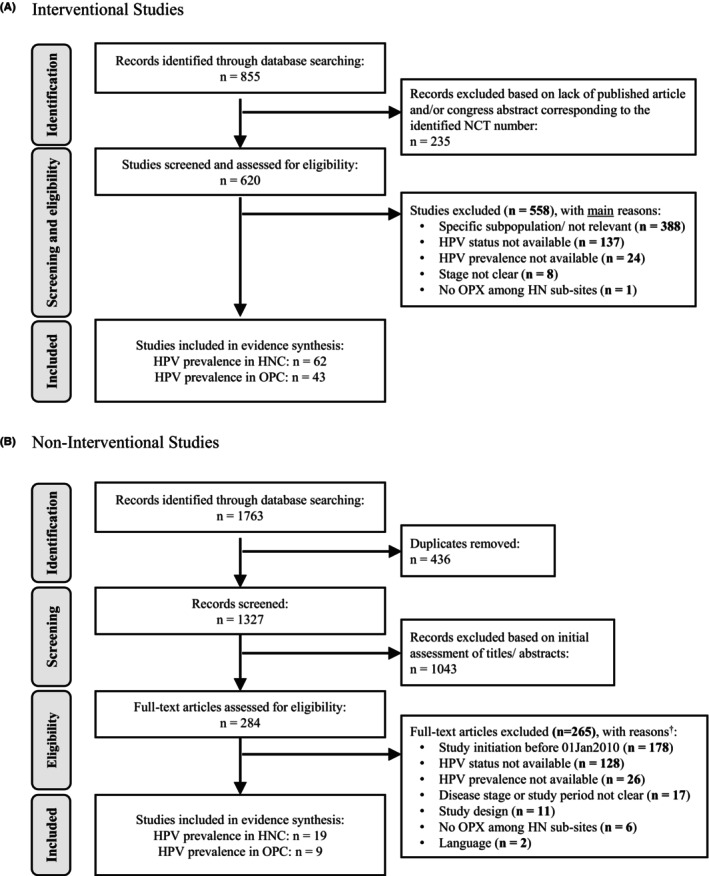
PRISMA diagrams for selection of (A) interventional studies and (B) non‐interventional studies. HN, head and neck; HNC, head and neck cancer; HPV, human papilloma virus; *n*, number of studies; OPC, oropharyngeal cancer; OPX, oropharynx. ^†^Number of excluded articles per reason does not add up to total number of excluded articles as many cases were excluded for more than one reason.

The search strategy identified a total of 2618 records, of which 855 corresponded to IS and 1763 to NIS. Following removal of duplicate records, records with lack of published articles and/or congress abstracts, and studies not fulfilling the PICOTS criteria, a total of 62 IS and 19 NIS were included in the evidence synthesis (Figure 1).

All included studies (*N* = 81) provided an HPV prevalence of LA and/or RM HNC captured between January 01, 2010 and December 31, 2020 and were used for addressing the primary study objective. Of the included studies, 43 IS and 9 NIS reported data on prevalence of HPV specifically for OPC, and were thus used to address the secondary outcome of interest.

Characteristics of the studies included in the evidence synthesis and outcomes of interest derived from each study are presented in Table 1 and Table S1. Of the IS, 42 (67.7%) were single‐arm and 20 (32.3%) were multi‐arm; of the latter 17 (27.5%) were randomized. Of the NIS, 13 (68.4%) were retrospective, 2 (10.5%) were prospective cohort studies, another 2 (10.5%) were cross‐sectional studies, and the remaining 2 were of a mixed cohort study design (10.5%). Sixty‐one (75.3%) of the included studies were single‐country studies conducted in Northern America, Europe, and Asia, 5 (6.2%) were multi‐country, single‐continent studies, and the remaining 15 (18.5%) were multi‐country, multi‐continent studies. Overall, the selected studies were conducted in 51 countries distributed in all continents (Table 1).

**TABLE 1:** Patients' characteristics and outcomes of interest in studies included in the evidence synthesis.

Study acronym or primary author (year)	Country	Median age (range)	Disease stage	HPV status detection methodology^e^	Subsites that HPV status was assessed	N_HNC_ enrolled	N_HNC_ HPV+	N_HNC_ HPV–	HPV prevalence (%) in HNC	N_OPC_ enrolled	OPC fraction (%) in HNC	N_OPC_ HPV+	HPV prevalence (%) in OPC
Interventional Studies													
NCT01045421^46^	CZ, FR, PL, USA	58 (52–65)	RM	IHC	Any site	55	14	16	25.5	n/a	n/a	10	n/a
NCT01126216^47^	DE	59 (36–80)	LA	p16	Any site	216	49	110	22.7	116	53.7	32	27.6
NCT01133678^48^	USA	57 (27–76)	LA	p16/ISH	OPX	94	59	n/a	62.8	71	75.5	59	83.1
NCT01172769^49^	DE	61.5 (42–79)	RM	DNA	Any site	40	4	20	10.0	15	37.5	3	20.0
NCT01195922^50^	USA	60^a^ (n/a)	LA	p16	Any site	16	8	8	50.0	8	50.0	6	75.0
NCT01218048^51^	USA	59 (31–93)	LA	n/a	Any site	29	10	19	34.5	11	37.9	9	81.8
NCT01255800^52^	USA	57 (n/a)	RM	ISH	Any site	9	5	3	55.6	n/a	n/a	n/a	n/a
NCT01345682^53^, ^54^	AR, AT, BE, BR, CH, CZ, DE, DK, ES, FR, GR, IL, IT, JP, MX, RU, SE, USA, ZA	n/a (n/a)	RM	p16	Any site	234^c^	35	199	15.0	80	34.2	23	28.8
NCT01379339^55^	USA	n/a (n/a)	LA	p16	OPX	26	9	2	34.6	13	50.0	9	69.2
NCT01412229^56^	USA	57^a^ (39–70)	LA	p16	OPX	38	17	5	44.7	25	65.8	17	68.0
NCT01417936^57^	BE, DE, FR	60 (42–87)	RM	PCR	Any site	26	1	20	3.8	13	50.0	n/a	n/a
NCT01437449^58^	USA	60 (24–80)	RM	p16	OPX	29	11	n/a	37.9	14	48.3	11	78.6
NCT01449201^59^	KR	60.5 (30–82)	RM	p16	Any site	48	8	26	16.7	11	22.9	n/a	n/a
NCT01458392^60^	USA	60.5 (45–78)	RM	n/a	Any site	46	21	19	45.7	21	45.7	n/a	n/a
NCT01468896^61^	USA	60^a^ (41–82)	RM	n/a	Any site	23	12	5	52.2	1	4.3	n/a	n/a
NCT01472653^62^	SI	57 (42–75)	LA	DNA/mRNA	OPX	39	8	18	20.5	30	76.9	8	26.7
NCT01566435^63^, ^64^	USA	57 (43–75)	LA	p16	OPX	30	17	n/a	56.7	18	60.0	17	94.4
NCT01577173^65^	AU, BE, BG, DE, ES, FR, HU, IT, RO, UK, USA	62.0 (28–84)	RM	qRT‐PCR	Any site	121	25	85	20.7	36	29.8	14	38.9
NCT01592721^66^	USA	66.5^a^ (50–81)	LA	p16/ISH	Any site	6	1	3	16.7	4	66.7	1	25.0
NCT01612351^67^	USA	57.5^a^ (41–77)	LA	p16 & PCR	OPX	40	17	n/a	42.5	30	75.0	17	56.7
NCT01696955^68^, ^69^	USA	60.5 & 63.6 (35–90)	RM	p16	n/a	78	31	47	39.7	n/a	n/a	31	n/a
NCT01716416^70^	USA	59 (38–72)	RM	p16	OPX	31	8	23	25.8	9	29.0	8	88.9
NCT01737008^71^	CA	53.5^a^ (48–66)	LA	p16	Any site	6	6	n/a	100.0	5	83.3	5	100.0
NCT01816984^72^	USA	61^a^ (47–73)	RM	p16 and/or PCR	n/a	12	5	4	41.7	9	75.0	5	55.6
NCT01836029^73^	USA	58 (23–81)	RM	p16/ISH/PCR	OPX	195	52	25	26.7	83	42.6	52	62.7
NCT01848834^74^, ^75^, ^76^ ^b^	IL, JP, KR, TW, USA	60 (20–84)	RM	p16	OPX	192	45	147	23.4	76	39.6	45	59.2
NCT01856478^77^	CN, EG, HK, IN, KR, PH, TH, TW	55.5 & 58.0 (27–83)	RM	p16	Any site	340	10	109	2.9	47	13.8	n/a	n/a
NCT01911598^78^	BE, USA	61 (29–82)	RM	qRT‐PCR	Any site	24	5	19	20.8	9	37.5	n/a	n/a
NCT01935921^79^	USA	n/a (n/a)	LA	n/a	OPX	18	7	3	38.9	10	55.6	7	70.0
NCT01946867^80^	ES, FR	78 (65–91)	LA	p16	OPX	19	6	1	31.6	13	68.4	6	46.2
NCT01969877^81^	SE	61 (33–77)	LA	p16	OPX	291	221	25	75.9	248	85.2	221	89.1
NCT02052960^82^, ^83^	BE, DE, ES, FR, IT, PL, RO	n/a (n/a)	RM	p16	n/a	240	32	n/a	13.3	n/a	n/a	n/a	n/a
NCT02105636^84^	AR, BR, CA, CH, DE, ES, FR, HK, IT, JP, KR, NL, TW, UK, USA	60 (28–83)	RM	p16	OPX	361	93	93	25.8	n/a	n/a	93	n/a
NCT02207530^85^	BE, CA, CZ, DE, ES, FR, GE, HU, KR, MY, TW, UK, USA	60 (24–84)	RM	p16/ISH/PCR	Any site	112	34	65	30.4	40	35.7	20	50.0
NCT02252042^86^	AU, BE, CA, CH, DE, ES, FR, HU, IE, IT, KR, LT, MX, NL, PL, PT, RU, SE, UK, USA	60 (54–66)	RM	p16	OPX	495	119	323	24.0	n/a	n/a	119	n/a
NCT02255097^87^	DK, NO, USA	61 (33–90)	RM	p16	OPX	171	37	131	21.6	100	58.5	37	37.0
NCT02268695^88^	DE, ES, FR	60 (55–64)	RM	ISH	OPX	503	34	146	6.8	180	35.8	34	18.9
NCT02274155^89^	USA	60^a^ (n/a)	LA	p16	OPX	17	6	9	35.3	9	52.9	6	66.7
NCT02277197^90^, ^91^	USA	n/a (n/a)	RM	p16	OPX	12	1	n/a	8.3	3	25.0	1	33.3
NCT02282371^92^	USA	60 (36–73)	LA	n/a	Any site	11	10	n/a	90.9	9	81.8	n/a	n/a
NCT02308072^93^	UK	60.81 (46.13–75.48)	LA	p16	OPX	16	5	2	31.3	7	43.8	5	71.4
NCT02319044^94^	AU, BE, CA, CZ, DE, ES, FR, GE, HU, IL, KR, MY, TW, UK, USA	61.0 (23–82)	RM	n/a	Any site	267	75	n/a	28.1	107	40.1	n/a	n/a
NCT02350712^95^	UK	53 (31–65)	RM	n/a	OPX	15	3	1	20.0	5	33.3	3	60.0
NCT02358031^96^	AR, AT, AU, BR, CA, CH, CL, CO, CZ, DE, DK, EE, ES, FI, GR, HK, HU, IL, IT, JP, LV, MX, MY, NL, NO, PE, PH, PL, RU, SE, SG, TH, TR, TW, UK, USA, ZA^d^	61.0 & 62.0 (54.5–68.0)	RM	p16	OPX	882	251	n/a	28.5	447	50.7	251	56.2
NCT02369874^97^	AR, AU, BE, BR, BG, CL, CZ, DE, ES, FR, GE, HR, HU, IL, IT, JP, KR, PL, RO, RS, RU, TW, UA, USA	60 (22–84)	RM	IHC/FISH/PCR [local standard procedures]	OPX	736	91	178	12.4	274	37.2	91	33.2
NCT02508389^98^	CA, USA	57 (30–84)	LA	p16	Any site	223	160	63	71.7	172	77.1	n/a	n/a
NCT02537223^99^	CA	59 (52–70)	LA	p16	OPX	9	7	n/a	77.8	9	100.0	7	77.8
NCT02538510^100^	USA	61 (33–86)	RM	p16	OPX	25	13	n/a	52.0	17	68.0	13	76.5
NCT02549742^101^	DK	67.5 (47–87)	RM	p16	Any site	26	3	22	11.5	4	15.4	1	25.0
NCT02573493^102^	USA	n/a (n/a)	LA	p16	OPX	80	46	34	57.5	57	71.3	46	80.7
NCT02586207^103^	USA	59.8 (36–81)	LA	ISH/p16	Any site	59	34	25	57.6	40	67.8	31	77.5
NCT02609503^104^	USA	63.1^a^ (39–86)	LA	p16	OPX	29	14	n/a	48.3	20	69.0	14	70.0
NCT02626000^105^	AT, AU, BE, CA, CH, ES, FR, GR, IT, UK, USA	62 (35–77)	RM	n/a	OPX	36	5	4	13.9	9	25.0	5	55.6
NCT02643056^106^	CH	60 (42–87)	RM	n/a	n/a	33	10	18	30.3	n/a	n/a	n/a	n/a
NCT02707588^107^, ^108^	FR	65 (n/a)	LA	p16	n/a	133	37	n/a	27.8	80	60.2	n/a	n/a
NCT02718820^109^	AT	63 (44–77)	RM	p16	OPX	22	4	n/a	18.2	9	40.9	4	44.4
NCT02764593^110^	USA	62 (n/a)	LA	p16	OPX	39	24	n/a	62.0	n/a	n/a	24	n/a
NCT02938273^111^	NL	69 (61–77)	Other	n/a	OPX	10	4	5	40.0	8	80.0	4	50.0
NCT02999087^112^	CH	61^a^ (40–78)	LA	p16	OPX	82	28	n/a	34.1	60	73.2	28	46.7
NCT03003637^113^	NL	n/a (n/a)	Other	n/a	n/a	32	1	31	3.1	n/a	n/a	n/a	n/a
NCT03370276^114^	USA	n/a (n/a)	RM	p16	OPX	45	22	4	48.9	26	57.8	22	84.6
NCT04397341^115^	CN	53 (28–69)	LA	p16	OPX	58	6	19	10.3	25	43.1	6	24.0
Non‐interventional Studies													
Bossi (2016)^116^	IT	n/a (n/a)	LA	n/a	OPX	55	18	7	32.7	25	45.5	18	72.0
Bossi (2019)^117^	IT	60 (52–68)	LA	n/a	OPX	129	47	12	36.4	59	45.7	47	79.7
Botticelli (2020)^118^	IT	67 (30–82)	RM	n/a	Any site	61	2	11	3.3	14	23.0	2	14.3
Byrne (2019)^119^	CA	63 (57–68)	Other	p16	Any site	109	33	37	30.3	52	47.7	n/a	n/a
Castelli (2019)^120^	FR	59 & 62^a^ (n/a)	LA	p16	Any site	237	38	102	16.0	163	68.8	n/a	n/a
de Ridder (2020)^121^	NL	n/a (n/a)	RM	p16	Any site	198	23	171	11.6	89	44.9	n/a	n/a
Galot (2020)^122^	BE	n/a (n/a)	RM	p16	OPX	39	5	17	12.8	22	56.4	5	22.7
Grünwald (2020)^123^	AU, BR, CA, DE, ES, IT, KR, TW, UK	60 (54–67)	RM	p16	OPX	733	35	21	4.8	221	30.2	35	15.8
Hilke (2020)^124^	DE	n/a (n/a)	LA	p16	Any site	20	5	14	25.0	14	70.0	5	35.7
Kim (2020)^125^	KR	57.8 & 47.4 (16–74)	RM	p16	Any site	15	5	7	33.3	n/a	n/a	n/a	n/a
Martens (2019)^126^	NL	66.4^a^ (41.0–90.1)	RM	n/a	Any site	28	8	16	28.6	n/a	n/a	n/a	n/a
Martens (2020)^127^	NL	62.3 & 63.3^a^ (57.3–69.3)	LA	n/a	Any site	174	65	109	37.4	125	71.8	n/a	n/a
Nadler (2019)^128^	USA	62 (32–87)	RM	n/a	Any site	325	70	56	21.5	219	67.4	n/a	n/a
Noij (2018)^129^	NL	59.2 (43–81)	Other	p16, DNA	Any site	82	39	31	47.6	63	76.8	n/a	n/a
Pitak‐Arnnop (2020)^130^	DE	67^a^ (n/a)	RM	p16, ISH, DNA	Any site	9^b^	4	5	44.4	n/a	n/a	n/a	n/a
Porter (2020)^131^	USA	63 (44–89)	RM	n/a	Any site	60	15	9	25.0	21	35.0	15	71.4
Smirk (2018)^132^	UK	63 (n/a)	RM	n/a	Any site	29	6	21	20.7	9	31.0	n/a	n/a
Sridharan (2018)^133^	USA	57 (20–89)	Other	p16, ISH, DNA	Any site	100	42	58	42.0	51	51.0	42	82.4
Velez (2018)^134^	USA	58.5 (27.9–81.5)	RM	p16	Any site	54	16	7	29.6	14	25.9	3	21.4

Abbreviations: DNA, deoxyribonucleic acid; FISH; fluorescent in situ hybridization; HNC, head and neck cancer; HPV, human papilloma virus; IHC, immunohistochemistry; ISH, in situ hybridization; LA, locally and regionally advanced; mRNA, messenger ribonucleic acid; N, number of patients; n/a, not available; OPC, oropharyngeal cancer; OPX, oropharynx; PCR, polymerase chain reaction; qRT‐PCR, quantitative reverse transcription PCR; RM, recurrent and/or metastatic. **Country codes:** AR, Argentina; AT, Austria; AU, Australia; BE, Belgium; BG, Bulgaria; BR, Brazil; CA, Canada; CH, Switzerland; CL, Chile; CN, China; CO, Colombia; CZ, Czech Republic; DE, Germany; DK, Denmark; EE, Estonia; EG, Egypt; ES, Spain; FI, Finland; FR, France; GE, Georgia; GR, Greece; HK, Hong Kong; HR, Croatia; HU, Hungary; IE, Ireland; IL, Israel; IN, India; IT, Italy; JP, Japan; KR, Republic of Korea; LT, Lithuania; LV, Latvia; MX, Mexico; MY, Malaysia; NL, Netherlands; NO, Norway; PE, Peru; PH, Philippines; PL, Poland; PT, Portugal; RO, Romania; RS, Serbia; RU, Russian Federation; SE, Sweden; SG, Singapore; SI, Slovenia; TH, Thailand; TR, Turkey; TW, Taiwan; UA, Ukraine; UK, United Kingdom; USA, United States of America; ZA, South Africa.

^a^ Mean.

^b^ For multiple applicable cohorts but with potentially overlapping data, the largest cohort was included.

^c^ For HPV prevalence evidence synthesis, data from the subgroup of patients volunteering for tumor biomarker analysis were used, as available in Cohen et al. (2017).^54^

^d^ This study included 17 sites that actively screened individuals but did not have any participants randomly allocated to study treatment.

^e^ In cases where marker or target was not specified (e.g., IHC rather than p16 by IHC), the detection methodology is recorded as reported in the source.

According to the disease stage of the included population, 31 (38.3%) studies were classified as LA HNC, 45 (55.6%) as RM HNC, and the remaining 5 (6.2%) as Other. The selected studies cumulatively included 9607 LA and/or RM HNC patients. Median patient age ranged from 47 to 78 years across studies (Table 1).

### 3.2 Prevalence of HPV in HNC

**FIGURE 2:**
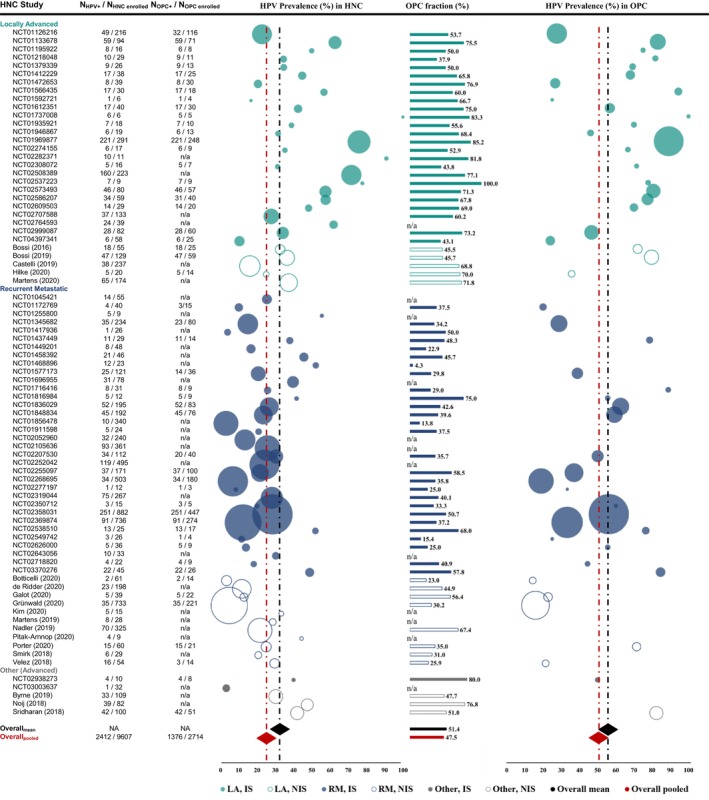
HPV prevalence in LA and RM HNC, OPC fraction, and HPV prevalence in LA and RM OPC. HNC, head and neck cancer; HPV, human papilloma virus; IS, interventional studies; LA, locally and regionally advanced; N, number of patients; NA, not applicable; n/a, not available; NIS, non‐interventional studies; OPC, oropharyngeal cancer; RM, recurrent and/or metastatic. Circle size corresponds to number of patients included in the study indicated, ranging from 6 to 882 patients across 81 studies in HNC, and from 3 to 447 patients across 52 studies in OPC. Overall HPV prevalence is provided as mean and pooled HPV prevalence across studies and depicted as a black and red diamond, respectively. Overall OPC fraction is provided as mean and pooled OPC fraction across studies as a black and red bar, respectively.

The proportion of HPV+ patients over HNC patients enrolled in each study, that is, HPV prevalence per study, and overall HPV prevalence are presented in Figure 2 and Table 2. The prevalence of HPV in HNC varied considerably across studies, ranging from 2.9% to 100.0%, with a mean value of 32.6%. To account for variations in sample size of each included study, the pooled HPV prevalence was also calculated across studies and was found to be 25.1%. In the IS (*n* = 62), the prevalence of HPV ranged from 2.9% to 100.0%, with a mean value of 34.5% and a pooled HPV prevalence of 27.1%; while in NIS (*n* = 19) the prevalence of HPV ranged from 3.3% to 47.6%, with a mean value of 26.5% and a pooled HPV prevalence of 19.4%. In a further analysis by disease stage and regardless of study design, prevalence of HPV was examined in the subgroups of patients with LA and RM, as these represent distinct disease phenotypes with different management approaches and survival outcomes. In LA HNC studies (*n* = 31) HPV prevalence ranged from 10.3% to 100.0% (mean 44.7%), with a pooled fraction of 44.0%, while in RM HNC studies (*n* = 45), HPV prevalence ranged from 2.9% to 55.6% (mean 24.3%), with a pooled fraction of 18.6%. Interestingly, the prevalence of HPV exceeded 50.0% in about one sixth of all studies included in the evidence synthesis.

**TABLE 2:** HPV prevalence in LA and RM HNC and OPC, and OPC fraction, per design and stage.

HPV prevalence in HNC							
	*n* studies	Mean	Median	Range (min, max)	N_HNC_ pts enrolled	N_HPV+ HNC_ pts	Pooled
Interventional Studies							
LA	26	47.6%	43.6%	10.3%–100.0%	1624	812	50.0%
RM	34	25.2%	23.7%	2.9%–55.6%	5484	1119	20.4%
Other	2	NA	NA	3.1%–40.0%	42	5	NA
**Overall**	**62**	**34.5%**	**30.4%**	**2.9%–100.0%**	**7150**	**1936**	**27.1%**
Non‐interventional Studies							
LA	5	29.5%	32.7%	16.0%–37.4%	615	173	28.1%
RM	11	21.4%	21.5%	3.3%–44.4%	1551	189	12.2%
Other	3	NA	NA	30.3%–47.6%	291	114	NA
**Overall**	**19**	**26.5%**	**28.6%**	**3.3%–47.6%**	**2457**	**476**	**19.4%**
Interventional and non‐interventional studies							
LA	31	44.7%	37.4%	10.3%–100.0%	2239	985	44.0%
RM	45	24.3%	23.4%	2.9%–55.6%	7035	1308	18.6%
Other	5	NA	NA	3.1%–47.6%	333	119	NA
**Overall**	**81**	**32.6%**	**29.6%**	**2.9%–100.0%**	**9607**	**2412**	**25.1%**

**OPC fraction in HNC**							
	**n**_**studies**_	**Mean**	**Median**	**Range (min, max)**	**N**_**HNC**_ **pts enrolled**	**N**_**OPC**_ **pts enrolled**	**Pooled**
Interventional Studies							
LA	25	65.8%	67.8%	37.9%–100.0%	1585	1090	68.8%
RM	27	38.3%	37.5%	4.3%–75.0%	4213	1645	39.0%
Other	1	NA	NA	NA	10	8	NA
**Overall**	**53**	**52.0%**	**50.0%**	**4.3%–100.0%**	**5808**	**2743**	**47.2%**
Non‐interventional Studies							
LA	5	60.4%	68.8%	45.5%–71.8%	615	386	62.8%
RM	8	39.2%	33.0%	23.0%–67.4%	1499	609	40.6%
Other	3	NA	NA	NA	291	166	NA
**Overall**	**16**	**49.4%**	**46.7%**	**23.0%–76.8%**	**2405**	**1161**	**48.3%**
Interventional and Non‐interventional Studies							
LA	30	64.9%	68.1%	37.9%–100.0%	2200	1476	67.1%
RM	35	38.5%	37.2%	4.3%–75.0%	5712	2254	39.5%
Other	4	NA	NA	NA	301	174	NA
**Overall**	**69**	**51.4%**	**50.0%**	**4.3%–100.0%**	**8213**	**3904**	**47.5%**

**HPV Prevalence in OPC**							
	**n**_**studies**_	**Mean**	**Median**	**Range (min, max)**	**N**_**OPC pts enrolled**_	**N**_**HPV+ OPC pts**_	**Pooled**
Interventional Studies							
LA	22	64.9%	70.0%	24.0%–100.0%	829	557	67.2%
RM	20	50.4%	52.8%	18.9%–88.9%	1436	643	44.8%
Other	1	ΝΑ	NA	ΝΑ	8	4	NA
**Overall**	**43**	**57.8%**	59.2%	**18.9%–100.0%**	**2273**	**1204**	**53.0%**
Non‐interventional Studies							
LA	3	62.5%	72.0%	35.7%–79.7%	98	70	71.4%
RM	5	29.1%	21.4%	14.3%–71.4%	292	60	20.5%
Other	1	ΝΑ	NA	ΝΑ	51	42	NA
**Overall**	**9**	**46.2%**	35.7%	**14.3%–82.4%**	**441**	**172**	**39.0%**
Interventional and Non‐interventional Studies							
LA	25	64.6%	70.0%	24.0%–100.0%	927	627	67.6%
RM	25	46.1%	44.4%	14.3%–88.9%	1728	703	40.7%
Other	2	ΝΑ	NA	ΝΑ	59	46	NA
**Overall**	**52**	**55.8%**	**57.9%**	**14.3%–100.0%**	**2714**	**1376**	**50.7%**

Abbreviations: HNC, head and neck cancer; HPV, human papilloma virus; LA, locally and regionally advanced; N pts, number of patients; *n*, number of studies; NA, not applicable; OPC, oropharyngeal cancer; RM, recurrent/metastatic.

### 3.3 Prevalence of HPV in OPC

In light of the increasing incidence of OPC reported in the literature, and the established role of HPV in OPC pathogenesis and prognosis, we assessed the proportion of HPV+ patients in the highly relevant subgroup of patients with LA and/or RM OPC. The proportion of patients with OPC among those with LA and/or RM HNC, referred to as the OPC fraction, is presented in Figure 2 and summarized in Table 2. The OPC fraction among studies with available HN subsite proportions (*n* = 69) ranged from 4.3% to 100.0%, with a mean of 51.4%. Based on pooled data, of the 8213 LA and/or RM HNC patients, 3904 had OPC, resulting in a pooled fraction of 47.5%. The mean (and pooled) fractions in IS (*n* = 53; range 4.3% to 100.0%) and NIS (*n* = 16; range 23.0% to 76.8%) were 52.0% (47.2%) and 49.4% (48.3%), respectively. Upon analysis by disease stage, the mean (and pooled) OPC fraction was 64.9% (67.1%) in LA HNC studies (*n* = 30; range 37.9% to 100.0%) and 38.5% (39.5%) in RM HNC studies (*n* = 35; ranging from 4.3% to 75.0%) (Table 2).

HPV prevalence in LA and/or RM OPC was available for 52 studies and ranged from 14.3% to 100.0%, with a mean value of 55.8% and a pooled fraction of 50.7%. HPV prevalence in LA and/or RM OPC ranged from 18.9% to 100.0% in IS, and from 14.3% to 82.4% in NIS with available data, with respective mean (and pooled) rates of 57.8% (53.0%) and 46.2% (39.0%). Upon analysis by disease stage, the mean (and pooled) HPV prevalence in LA OPC studies was 64.6% (67.6%), ranging from 24.0% to 100.0%, while in RM OPC studies it was 46.1% (40.7%), ranging from 14.3% to 88.9% (Table 2).

### 3.4 Geographic distribution of HPV prevalence

To gain insight into the availability of published data on the prevalence of HPV across geographical regions, as well as to qualitatively assess potential variations among countries or regions, the geographic distribution of HPV prevalence was addressed as an exploratory objective. Of the 54 countries where the studies included in the analysis of the present review were conducted, 29 were located in Europe, 13 in Asia (including Hong Kong as territory of China), 5 in Southern America, 2 countries each in Northern America and Africa, and 1 country each in Central America and Oceania. Τhe following countries were included in more than ten studies each: United States of America (USA) (44 studies), Germany (16), France (15), Spain (13), Italy (12), Belgium (11), Canada (11), and the United Kingdom (11) (Table 1). Thus, although studies with published data on HPV prevalence in LA and RM HNC through the last decade display a wide geographic distribution, several geographic regions are underrepresented in the literature and further studies would be needed to more accurately capture the global epidemiological picture.

The prevalence of HPV in LA and RM HNC and OPC is summarized per geographical region in Table 3 and Figure S1, while it is also presented per disease stage in Figure S2. Based on the geographic regions included, studies can be broadly divided into those conducted in a single continent and those conducted in multiple continents. In single‐continent HNC studies conducted in Northern America (*n* = 34), the prevalence of HPV ranged from 8.3% to 100.0%; in Europe (*n* = 29) from 3.1% to 75.9%; in Eastern Asia (*n* = 3) from 10.3% to 33.3%. The mean (and pooled) prevalence of HPV among single‐continent studies conducted in Northern America was 46.0% (42.1%), followed by 24.7% (25.3%) in Europe, and 20.1% (15.7%) in Eastern Asia. Studies conducted in Europe were also grouped into those conducted in Northern Europe, Southern Europe, Western Europe, or multiple European regions (including Western, Central/Eastern, and Southern Europe) based on data availability. The respective mean (and pooled) HPV prevalence was 31.9% (63.1%), 23.2% (26.4%), 24.3% (23.5%), and 17.2% (9.4%). In studies conducted in multiple continents (*n* = 15) the prevalence of HPV ranged from 2.9% to 30.4%, and the mean (and pooled) prevalence of HPV was 19.8% (18.4%).

**TABLE 3:** HPV prevalence in LA and RM HNC and OPC, and OPC fraction, per geographic region.

HPV Prevalence in HNC							
	*n* studies	Mean	Median	Range (min, max)	N_HNC_ pts enrolled	N_HPV+ HNC_ pts	Pooled
**Northern America**	34	46.0%	43.6%	8.3%–100.0%	1923	809	42.1%
**Europe**	29	24.7%	22.7%	3.1%–75.9%	2804	710	25.3%
Northern Europe	5	31.9%	20.7%	11.5%–75.9%	377	238	63.1%
Southern Europe	4	23.2%	26.6%	3.3%–36.4%	284	75	26.4%
Western Europe	17	24.3%	25.0%	3.1%–47.6%	1381	325	23.5%
Multiple European regions^a^	3	17.2%	13.3%	6.8%–31.6%	762	72	9.4%
**Eastern Asia**	3	20.1%	16.7%	10.3%–33.3%	121	19	15.7%
**Multiple continents**	15	19.8%	21.6%	2.9%–30.4%	4759	874	18.4%
**OPC Fraction in HNC**							
	***n* studies**	**Mean**	**Median**	**Range (min, max)**	**N**_**HNC**_ **pts enrolled**	**N**_**OPC**_ **pts enrolled**	**Pooled**
**Northern America**	**31**	**56.9%**	**57.8%**	**4.3%–100.0%**	**1797**	**1051**	**58.5%**
**Europe**	**24**	**53.7%**	**51.9%**	**15.4%–85.2%**	**2462**	**1371**	**55.7%**
Northern Europe	5	41.7%	33.3%	15.4%–85.2%	377	273	72.4%
Southern Europe	4	47.8%	45.6%	23.0%–76.9%	284	128	45.1%
Western Europe	13	60.3%	60.2%	37.5%–80.0%	1279	777	60.8%
Multiple European regions^a^	2	52.1%	52.1%	35.8%–68.4%	522	193	37.0%
**Eastern Asia**	**2**	**33.0%**	**33.0%**	**22.9%–43.1%**	**106**	**36**	**34.0%**
**Multiple continents**	**12**	**36.0%**	**36.5%**	**13.8%–58.5%**	**3848**	**1446**	**37.6%**
**HPV Prevalence in OPC**							
	***n***_**studies**_	**Mean**	**Median**	**Range (min, max)**	**N**_**OPC**_ **pts enrolled**	**N**_**HPV+ OPC**_ **pts**	**Pooled**
**Northern America**	25	70.0%	75.0%	21.4%–100.0%	577	423	73.3%
**Europe**	17	44.1%	44.4%	14.3%–89.1%	829	426	51.4%
Northern Europe	4	61.4%	65.7%	25.0%–89.1%	264	230	87.1%
Southern Europe	4	48.2%	49.3%	14.3%–79.7%	128	75	58.6%
Western Europe	7	35.3%	35.7%	20.0%–50.0%	244	81	33.2%
Multiple European regions^a^	2	32.5%	32.5%	18.9%–46.2%	193	40	20.7%
**Eastern Asia**	1	NA	NA	NA	25	6	24.0%
**Multiple continents**	9	41.6%	38.9%	15.8%–59.2%	1283	521	40.6%

Abbreviations: HPV, human papilloma virus; HNC, head and neck cancer; N pts, number of patients; *n*, number of studies; NA, not applicable; OPC, oropharyngeal cancer.

^a^ The category of multiple European regions includes multi‐country studies conducted in Europe. These studies were conducted in Western, Central/Eastern, and Southern Europe for HNC, and Southern and Western Europe for OPC.

Among studies with available HN subsite proportions (regardless of HPV status), mean (and pooled) OPC fraction was 56.9% (58.5%), 53.7% (55.7%), 33.0% (34.0%), and 36.0% (37.6%) in studies conducted in Northern America (*n* = 31), Europe (*n* = 24), Eastern Asia (*n* = 2), and multiple continents (*n* = 12), respectively (Table 3). Moreover, based on the proportion of HPV+ OPC patients in studies with available data, the mean (and pooled) prevalence of HPV in LA and RM OPC was 70.0% (73.3%) in studies conducted in Northern America (*n* = 25); 44.1% (51.4%) in studies conducted in Europe (*n* = 17), and 41.6% (40.6%) in studies conducted in multiple continents (*n* = 9). In the only single‐country study conducted in Eastern Asia, the prevalence of HPV in LA and RM OPC was 24.0% (Table 3). Within Europe, the mean (and pooled) prevalence of HPV in LA and RM OPC was 61.4% (87.1%) in Northern Europe, 48.2% (58.6%) in Southern Europe, 35.3% (33.2%) in Western Europe, and 32.5% (20.7%) in multiple European regions (including Southern and Western Europe). Taken together, the above data illustrate high rates of HPV prevalence in LA and RM HNC and OPC across different geographical regions.

### 3.5 HPV detection techniques

In the absence of HPV diagnostic tests with regulatory approval for HNC over the examined period, and given that HPV testing is generally recommended for all newly diagnosed OPSCC but is not warranted for the other HNC types, the present review aimed to capture HPV detection techniques utilized in the included studies. HPV detection techniques are retrieved and analyzed as reported by the authors in the publications. Information on reported HPV detection assays across the included HNC studies are presented in Figure S3. In total, HPV status was assessed in any HN anatomical site in 37 studies (45.7%), in OPX only in 38 studies (46.9%) while 6 studies (7.4%) did not provide information on the site examined. With respect to specific methodologies, of the 81 studies, 47 (58.0%) reported using a p16^INK4a^‐based method, 2 studies (2.5%) employed quantitative reverse transcription‐polymerase chain reaction (qRT‐PCR), 2 studies (2.5%) employed in situ hybridization (ISH), while in 1 study each the detection method was referred to as DNA testing, PCR (qRT‐PCR), and immunohistochemistry (IHC). Eight studies (9.9%) reported using multiple detection techniques to determine HPV status at a cohort level, even though at a patient level HPV status could also have been derived solely based on a single technique. For the remaining 19 (23.5%) studies the authors did not provide any relevant information. HPV detection methods are also presented for IS and NIS, by disease stage, and site examined in Figure S3. Irrespective of grouping, “p16‐based” detection methodologies were the most frequently reported across studies.

### 3.6 HPV prevalence in OPC using solely a p16‐based method

Considering that p16 overexpression is generally used as a surrogate marker for the presence of HPV in OPSCC and the recommendation for p16 testing in OPSCC clinical management,^14^, ^15^ a supplementary analysis was performed by isolating the studies reporting solely a p16‐based method for HPV testing and having available results in OPC. In total, 30 studies were included in this analysis (26 IS and 4 NIS; 16 LA and 14 RM), with prevalence of HPV ranging from 15.8% to 100.0% and a mean (and pooled) HPV prevalence of 57.2% (52.6%) (Figure S4), further supporting the main outcomes of this evidence synthesis.

### 3.7 Distribution of HPV prevalence by number of enrolled HNC patients

**FIGURE 3:**
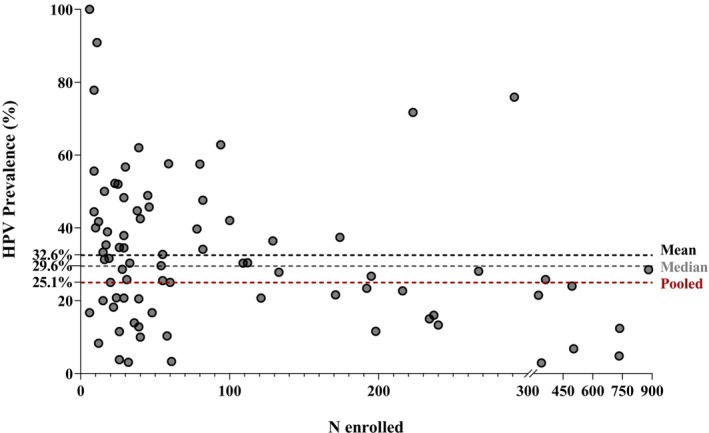
HPV prevalence by number of HNC cases in each study. HPV, human papilloma virus; N, number of enrolled HNC patients. HPV prevalence from studies included in the evidence synthesis is plotted against each study's size. To visualize distribution of studies around the overall HPV prevalence and potential effect of sample size, estimated mean, median, and pooled HPV prevalence across studies are provided in dotted lines.

As a means to evaluate the potential effect of variations across individual sample sizes on the primary outcome of overall prevalence of HPV in LA and RM HNC, the prevalence of HPV reported for each included study was plotted against the respective sample size (Figure 3). No obvious asymmetry was observed around the calculated overall mean HPV prevalence. Based on this distribution, no apparent bias in the estimation of the study primary outcome arising from sample size can be inferred.

## 4 DISCUSSION

Contemporary data on HPV burden in advanced HNC are needed considering that available published reviews are not only outdated but also lack disease stage‐specific estimates. Thus, the present SLR aimed to fill this gap through a systematic review of published data on the prevalence of HPV in LA and RM HNC captured over the last decade. Using specific selection criteria, 81 studies were identified, reporting data from numerous countries and covering available literature from 2010 to 2020. The results revealed a considerable HPV prevalence in LA and RM HNC and OPC across different regions. Numerically highest rates were reported in the LA setting, as well as in the regions of Northern America and Northern Europe. In addition, the data uncover substantial variation in HPV prevalence among studies, as well as in HPV testing methodologies.

The primary outcome of “ALARM,” prevalence of HPV in LA and RM HNC, was first retrieved from each study as the minimum proportion of HPV+ cases, unadjusted for HPV status data availability. In this manner, selection bias which would otherwise arise from increased testing of a certain type of cases as per the investigator's clinical judgment was minimized to the extent possible. The average HPV prevalence in the LA and RM setting of HNC across studies was estimated at 32.6%, while the pooled HPV prevalence, derived from the sum of HPV+ patients over the sum of enrolled HNC patients in all studies was 25.1%. Other reviews have estimated HPV prevalence in HNC (any stage) at 22%–35%, though based on literature data preceding 2008.^31^, ^32^, ^33^, ^34^ A more recent review of studies published between 2003 and 2014 estimated the mean percentage of HPV+ HNSCC patients at 32.9%,^5^ which was however derived from patients with available HPV status data only, regardless of disease stage. When we plotted reported HPV prevalence against the size of the population in each study, an apparently even distribution around the mean was observed, suggesting there was no significant bias due to outliers representing very small or very large sample sizes. On the other hand, data are slightly skewed in relation to the pooled estimate, which could derive from the large proportion of patients with unknown HPV status in some studies. Indeed, the top 10 studies with highest sample size contributed data for 51% of the total number of patients included in this SLR (4933 out of 9607) but more than half of those patients had unknown HPV status. This indicates that the estimated pooled prevalence of HPV is most likely underestimated, and also explains why the pooled estimate is smaller than the mean. As the latter does not account for missing data either, and given that previous literature suggests that more than 70% of HPV+ HNC patients have LA and/or RM HNC,^5^, ^31^ the mean HPV prevalence of 32.6% estimated here is probably lower than the actual proportion of HPV+ HNC fraction, further highlighting the important contributing role of HPV in LA and RM HNC.

Evidence to date suggests that HPV prevalence is higher among OPC than other HN subsites.^5^, ^6^, ^7^, ^36^, ^135^, ^136^ Importantly, though the prognostic impact of HPV status in non‐OPC HNC is unclear,^24^, ^25^, ^26^ HPV infection is an established prognostic indicator of treatment outcome in OPC.^14^, ^15^, ^16^ Hence, findings of the overall HPV prevalence herein should be interpreted in the context of the contribution of OPC, while special attention should also be drawn to HPV+ estimates within this highly relevant HNC subgroup.

Irrespective of HPV status, almost half of the overall population in “ALARM” had OPC. The etiological role of HPV in oropharyngeal carcinogenesis is widely recognized^4^ and p16 testing is recommended for OPSCC management,^12^, ^14^, ^21^ hence HPV testing is expected to be more frequent among OPC patients. Indeed, in “ALARM” in almost half of the studies (47%), HPV status was assessed in OPX only, reflecting current clinical practice. Though the study has not been designed to make any such comparisons, the prevalence of HPV is numerically higher in OPC than in not site‐specific HNC, consistent with the published literature.^5^, ^6^, ^7^ Specifically in LA and RM OPC, our data report the mean HPV prevalence at 55.8%, with a pooled prevalence of 50.7%, which is almost double than the overall rate in LA and RM HNC. The prevalence of HPV in LA and RM OPC is close to that reported in a previous review, which showed a mean of 49.9% HPV‐positive OPSCC, most of which comprised of stage III/IV disease (85.7%).^5^ Furthermore, in our supplementary analysis of HPV status in OPC tested using solely a p16‐based method which is the guideline recommended method,^12^, ^13^, ^21^, ^22^, ^23^ the mean prevalence of HPV was 57.2%, further supporting the robustness of the main study outcomes. As OPSCC has been increasing worldwide over the past years,^9^, ^35^, ^136^, ^137^, ^138^ our results reinforce the substantial contribution of HPV+ OPC to the overall burden especially in LA and RM HNC.

In “ALARM,” HPV prevalence was also investigated by disease stage. Estimates of HPV prevalence in LA HNC were numerically higher than in RM HNC (mean: 44.7% vs. 24.3%; pooled: 44.0% vs. 18.6%). This should also be interpreted taking into account the OPC fraction and HPV status availability, both of which were higher among LA than in RM patients (65% vs. 39% and at least 70% vs. 48%, respectively). Nevertheless, a numerically higher HPV prevalence was also noted in LA than in RM OPC patients (mean: 64.6% vs. 46.1%; pooled: 67.6% vs. 40.7%), which might be worth investigating further, especially considering that a large proportion of HNC cases are either diagnosed at LA stage or experience disease recurrence from LA to RM stage, and that HPV+ cancers are considered to have better prognosis.^28^, ^29^, ^30^

Previous literature has shown that the incidence of HNC anatomical subsites classified as a proxy for HPV infection, including the oropharynx, has been rising and an increased OPSCC HPV prevalence has been observed over the years especially in Northern America and Northern Europe.^9^, ^35^, ^136^, ^137^, ^138^ Moreover, HPV prevalence in OPC was higher in more developed regions than in developing countries.^6^, ^34^, ^138^ This is also reflected by the outcomes of the present review in terms of the heterogeneous geographic distribution of HPV prevalence being highest in studies conducted in Northern America and Northern Europe. The observed patterns could be attributable to several factors, such as HPV epidemiology which shows variation by ethnicity and gender, and is linked to lifestyle behaviors.^136^, ^139^, ^140^, ^141^, ^142^, ^143^, ^144^ Nevertheless, regardless of the regional variations in HPV prevalence and the factors that could contribute to the observed patterns, the results of the present study demonstrate that, though ranking lower in terms of prevalence than Northern America and Northern Europe, other parts of Europe and the globe, in general, have substantial rates of HPV+ HNC. These findings suggest that the need to implement preventive measures against HPV is imperative worldwide, and not only in the countries with the highest HPV burden.

The results of “ALARM” reveal a considerable inconsistency in the availability of HPV prevalence data across countries and continents, with many parts of the world being underrepresented. In particular, countries in continents other than Northern America or Europe were mainly represented by the group of multi‐continent studies, which could not be stratified further as relevant publications did not contain the required level of detail. Thus, there is a dearth of information on the HPV burden in those countries. This is in line with previous literature on specific ethnic groups which seems to be lacking in terms of population‐based studies.^145^ Altogether these observations suggest a need for further investigation, in order to represent all geographical regions in the literature and better assess the burden of this disease.

Another factor that could be contributing to the variation in reported HPV prevalence across studies is the heterogeneity in HPV detection assays. Many of the studies included in the present SLR reported p16‐based detection as the main assay (63% of studies, including four studies which used multiple techniques) but differences in the exact methodology, including specimen storage methods, p16‐positivity threshold used to define HPV status, and source of result (e.g., medical records archived or freshly collected samples) cannot be excluded. Furthermore, methodology was not specified for one fourth of the studies of the evidence synthesis, uncovering significant literature gaps. As depicted in the present review, clinical practice usually relies on a single technique for HPV status assessment, even though each technique has its limitations. In OPC, p16 testing is generally the preferred method of HPV detection, yet for other HNC sites there is no clear guidance on the HPV testing methodology.^12^, ^18^, ^146^ Novel diagnostic algorithms for the detection of HPV‐driven HNC are being examined, with the combined use of HPV‐DNA testing followed by p16 IHC having shown high concordance rates with E6*I mRNA detection and proposed to be helpful in OPC and oral cavity cancers.^147^ To improve the precision of HPV burden estimates, standardization of HPV detection is necessary.

Methodological limitations are presumably also a source of bias in the present analysis, as in non‐OPC HNC cases where HPV prevalence has been derived solely based on p16 overexpression, the estimates may not be accurate. Along this line, in the context of the primary outcome, the HPV prevalence, is possibly underestimated, as a result of the large proportion of patients with unknown HPV status. In any case, such limitations of the present review mainly derive from limitations of the individual studies included. In addition, certain limitations are due to the selection criteria applied in the present literature search, such as the exclusion of studies written in languages other than English, studies published in report format, for example, on government websites or studies that did not specify any study period or cancer stage. The above criteria may impact on the representativeness of the outcomes, but were employed as a method to ensure quality of included data. It should also be noted this SLR was designed to provide descriptive insight into the relevant literature from a qualitative point of view, including all studies that met a minimum set of criteria, with no restrictions in geographic location or patient eligibility (i.e., target indication, line of therapy, histology, or HN subsite) which increase the generalizability of the present findings. The latter is further enhanced by the fact that overlapping data have been avoided to the extent feasible based on geographic location, site, period of enrollment, and eligibility criteria in order to represent unique cases of HNC.

The results of the present literature search indicate a substantial proportion of HPV+ patients among LA and RM HNC patients in the last decade, which merits consideration particularly in light of increased awareness campaigns and preventive measures availability. HPV vaccines are effective in protecting against high‐risk HPV types in women and men.^135^, ^148^, ^149^, ^150^, ^151^, ^152^ In Europe, most countries recommend HPV vaccination, with many of them having introduced gender‐neutral HPV vaccination.^153^, ^154^, ^155^ HPV prevalence estimates can inform policy decisions and justify strategies to aim for higher levels of HPV vaccination coverage as well as ensure gender neutral vaccination for adolescents, timely catch‐up programs, and the possibility to vaccinate adults. Such measures are anticipated to prevent a significant proportion of LA and/or RM HNC especially in regions with a very high burden of HPV‐attributable HNC.

## 5 CONCLUSIONS

This SLR is the first review on HPV burden, which focused on LA and RM HNC and reported results from the last decade (2010–2020). More than 80 studies provided information on HPV status demonstrating that a substantial HPV burden exists with at least one in four HNC cases being HPV+ and at least half of OPC cases contributing to this proportion. The proportion of HPV+ cases was considerable in most regions examined, and highest in Northern America and Northern Europe, with at least one in three LA and/or RM HNC cases being HPV+. More quality data are however needed for a better representation of geographic diversity, and implementation of homogeneous HPV detection methodologies is necessary to allow for more precise HPV burden estimation. Nevertheless, the results of this evidence synthesis come to reinforce the significant role of HPV in LA and RM HNC disease with a considerable proportion of LA and RM HNC cases being potentially preventable, highlighting the potential benefit from increasing HPV immunization coverage.

## AUTHOR CONTRIBUTIONS

**Sofia Agelaki:** Conceptualization (equal); writing – review and editing (lead). **Ioannis Boukovinas:** Conceptualization (supporting); writing – review and editing (equal). **Ilias Athanasiadis:** Conceptualization (supporting); writing – review and editing (equal). **Georgios Trimis:** Conceptualization (equal); formal analysis (equal); investigation (equal); methodology (equal); supervision (equal); visualization (equal); writing – review and editing (equal). **Ioannis Dimitriadis:** Conceptualization (equal); writing – review and editing (equal). **Lazaros Poughias:** Conceptualization (supporting); writing – review and editing (equal). **Edith Morais:** Conceptualization (supporting); writing – review and editing (equal). **Ugne Sabale:** Conceptualization (supporting); writing – review and editing (equal). **Goran Bencina:** Conceptualization (equal); writing – review and editing (equal). **Charalampos Athanasopoulos:** Conceptualization (lead); formal analysis (lead); investigation (lead); methodology (lead); supervision (lead); visualization (lead); writing – original draft (lead).

## FUNDING INFORMATION

This work was funded by Merck Sharp & Dohme LLC, a subsidiary of Merck & Co, Inc., Rahway, NJ, USA.

## CONFLICT OF INTEREST STATEMENT

**Sofia Agelaki** is a member of the executive board of the Hellenic Society of Medical Oncology, has served as an investigator in clinical studies for MSD Greece, has received consulting fees for participating in Expert Input Forums for MSD Greece, and has received lecture honoraria by MSD Greece. **Ioannis Boukovinas**, former president of the Hellenic Society of Medical Oncology, has served as an investigator in clinical studies for MSD Greece, has received consulting fees for participating in Advisory Boards for MSD Greece, has received lecture honoraria by MSD Greece, and has received support for attending international congress from MSD Greece. **Ilias Athanasiadis** has served as an investigator in clinical studies for MSD Greece, has received consulting fees for participating in Advisory Boards for MSD Greece, has received lecture honoraria by MSD Greece, and has received support for attending international congress from MSD Greece. **Georgios Trimis**, **Ioannis Dimitriadis**, **Lazaros Poughias**, and **Charalampos Athanasopoulos** are employees of MSD Greece and own stock in Merck & Co., Inc., Rahway, NJ, US. **Edith Morais** is an employee of MSD France and owns stock in Merck & Co., Inc., Rahway, NJ, US. **Sabale Ugne** is an employee of MSD Sweden and owns stock in Merck & Co., Inc., Rahway, NJ, US. **Goran Bencina** is an employee of MSD Spain and owns stock in Merck & Co., Inc., Rahway, NJ, US.

## Supporting information


**Data S1:** Supporting information.

## Data Availability

The data that supports the findings of this study are included in the article/supplementary material. Data sharing is not applicable as no new data were created or analyzed in this study.
