# Supplementary material for: A systematic literature review of the human papillomavirus prevalence in locally and regionally advanced and recurrent/metastatic head and neck cancers through the last decade: The “ALARM” study
Source: Cancer Med. 2024 Jan 21;13(3):e6916. doi: 10.1002/cam4.6916 (PMC10905345; doi:10.1002/cam4.6916)
Supplement: Supplementary file 1 — Data S1: Supporting information. [file CAM4-13-e6916-s001.docx]

# Supporting Information

**Supplementary Data S1.** Preferred Reporting Items for Systematic Reviews and Meta-Analyses (PRISMA) Checklist.

**Supplementary Data S2.** Search strategy

**Supplementary Table S1.** Characteristics of studies included in the evidence synthesis.

**Supplementary Figure S1.** Summary of HPV prevalence in LA and RM (A) HNC and (B) OPC per geographic region.

**Supplementary Figure S2.** HPV prevalence in LA and RM (A) HNC and (B) OPC per region and disease stage.

**Supplementary Figure S3.** HPV detection methodologies, (A) overall and (B) by study design, disease stage and anatomical site of assessment

**Supplementary Figure S4.** HPV prevalence in advanced OPC tested for HPV positivity using solely a p16-based assay

## Supplementary Data S1. Preferred Reporting Items for Systematic Reviews and Meta-Analyses (PRISMA) Checklist.

The PRISMA statement is provided below based on guidelines published in 2020 ^1^ and available at <http://prisma-statement.org/PRISMAStatement/Checklist>.

| **Section and Topic** | **Item #** | **Checklist item** | **Starts on page** |
| --- | --- | --- | --- |
| **TITLE** | | |  |
| Title | 1 | Identify the report as a systematic review. | 1 |
| **ABSTRACT** | | |  |
| Abstract | 2 | See the PRISMA 2020 for Abstracts checklist. | 3 |
| **INTRODUCTION** | | |  |
| Rationale | 3 | Describe the rationale for the review in the context of existing knowledge. | 4 |
| Objectives | 4 | Provide an explicit statement of the objective(s) or question(s) the review addresses. | 4 |
| **METHODS** | | |  |
| Eligibility criteria | 5 | Specify the inclusion and exclusion criteria for the review and how studies were grouped for the syntheses. | 5 |
| Information sources | 6 | Specify all databases, registers, websites, organisations, reference lists and other sources searched or consulted to identify studies. Specify the date when each source was last searched or consulted. | 5 |
| Search strategy | 7 | Present the full search strategies for all databases, registers and websites, including any filters and limits used. | 5 |
| Selection process | 8 | Specify the methods used to decide whether a study met the inclusion criteria of the review, including how many reviewers screened each record and each report retrieved, whether they worked independently, and if applicable, details of automation tools used in the process. | 6 |
| Data collection process | 9 | Specify the methods used to collect data from reports, including how many reviewers collected data from each report, whether they worked independently, any processes for obtaining or confirming data from study investigators, and if applicable, details of automation tools used in the process. | 6 |
| Data items | 10a | List and define all outcomes for which data were sought. Specify whether all results that were compatible with each outcome domain in each study were sought (e.g. for all measures, time points, analyses), and if not, the methods used to decide which results to collect. | 6 |
|  | 10b | List and define all other variables for which data were sought (e.g. participant and intervention characteristics, funding sources). Describe any assumptions made about any missing or unclear information. | 6 |
| Study risk of bias assessment | 11 | Specify the methods used to assess risk of bias in the included studies, including details of the tool(s) used, how many reviewers assessed each study and whether they worked independently, and if applicable, details of automation tools used in the process. | 7 |
| Effect measures | 12 | Specify for each outcome the effect measure(s) (e.g. risk ratio, mean difference) used in the synthesis or presentation of results. | 6 |
| Synthesis methods | 13a | Describe the processes used to decide which studies were eligible for each synthesis (e.g. tabulating the study intervention characteristics and comparing against the planned groups for each synthesis (item #5)). | 6 |
|  | 13b | Describe any methods required to prepare the data for presentation or synthesis, such as handling of missing summary statistics, or data conversions. | 6 |
|  | 13c | Describe any methods used to tabulate or visually display results of individual studies and syntheses. | 6 |
|  | 13d | Describe any methods used to synthesize results and provide a rationale for the choice(s). If meta-analysis was performed, describe the model(s), method(s) to identify the presence and extent of statistical heterogeneity, and software package(s) used. | 6 |
|  | 13e | Describe any methods used to explore possible causes of heterogeneity among study results (e.g. subgroup analysis, meta-regression). | N/A |
|  | 13f | Describe any sensitivity analyses conducted to assess robustness of the synthesized results. | N/A |
| Reporting bias assessment | 14 | Describe any methods used to assess risk of bias due to missing results in a synthesis (arising from reporting biases). | N/A |
| Certainty assessment | 15 | Describe any methods used to assess certainty (or confidence) in the body of evidence for an outcome. | N/A |
| **RESULTS** | | |  |
| Study selection | 16a | Describe the results of the search and selection process, from the number of records identified in the search to the number of studies included in the review, ideally using a flow diagram. | 7 |
|  | 16b | Cite studies that might appear to meet the inclusion criteria, but which were excluded, and explain why they were excluded. | 7 |
| Study characteristics | 17 | Cite each included study and present its characteristics. | 7 |
| Risk of bias in studies | 18 | Present assessments of risk of bias for each included study. | N/A |
| Results of individual studies | 19 | For all outcomes, present, for each study: (a) summary statistics for each group (where appropriate) and (b) an effect estimate and its precision (e.g. confidence/credible interval), ideally using structured tables or plots. | 7 |
| Results of syntheses | 20a | For each synthesis, briefly summarise the characteristics and risk of bias among contributing studies. | N/A |
|  | 20b | Present results of all statistical syntheses conducted. If meta-analysis was done, present for each the summary estimate and its precision (e.g. confidence/credible interval) and measures of statistical heterogeneity. If comparing groups, describe the direction of the effect. | N/A |
|  | 20c | Present results of all investigations of possible causes of heterogeneity among study results. | N/A |
|  | 20d | Present results of all sensitivity analyses conducted to assess the robustness of the synthesized results. | N/A |
| Reporting biases | 21 | Present assessments of risk of bias due to missing results (arising from reporting biases) for each synthesis assessed. | N/A |
| Certainty of evidence | 22 | Present assessments of certainty (or confidence) in the body of evidence for each outcome assessed. | N/A |
| **DISCUSSION** | | |  |
| Discussion | 23a | Provide a general interpretation of the results in the context of other evidence. | 11 |
|  | 23b | Discuss any limitations of the evidence included in the review. | 12 |
|  | 23c | Discuss any limitations of the review processes used. | 13 |
|  | 23d | Discuss implications of the results for practice, policy, and future research. | 12 |
| **OTHER INFORMATION** | | |  |
| Registration and protocol | 24a | Provide registration information for the review, including register name and registration number, or state that the review was not registered. | 5 |
|  | 24b | Indicate where the review protocol can be accessed, or state that a protocol was not prepared. | 5 |
|  | 24c | Describe and explain any amendments to information provided at registration or in the protocol. | N/A |
| Support | 25 | Describe sources of financial or non-financial support for the review, and the role of the funders or sponsors in the review. | 1 |
| Competing interests | 26 | Declare any competing interests of review authors. | 1 |
| Availability of data, code and other materials | 27 | Report which of the following are publicly available and where they can be found: template data collection forms; data extracted from included studies; data used for all analyses; analytic code; any other materials used in the review. | 2 |

## Supplementary Data S2. Search strategy.

**Summary of search results for interventional and non-interventional studies**

| **Type of study** | **Database** | **Search date** | **Number of Hits** |
| --- | --- | --- | --- |
| Interventional | Clinical trials.gov | 19 March 2021 | **855** |
| Non-interventional | MEDLINE via Pubmed | 19 March 2021 | 832 |
| Non-interventional | Embase | 19 March 2021 | 931 |
| Non-interventional | **Total**** |  | **1,763** ^†^ |

^†^  Before duplicate removal.

**Detailed search strategy for interventional studies**

| **Key words** | | **Results** |
| --- | --- | --- |
| **1.** | Head and Neck | 6,469 |
| **2.** | #1 AND Local OR Regional OR Advanced OR Recurrent OR Metastatic | 3,946 |
| **3.** | #2 AND Filters: Phase 1, 2, 3 | 2,750 |
| **4.** | #3 AND Filters: Active, not recruiting, Completed, Unknown status Studies | 1,588 |
| **5.** | **#4 AND Start date on or after 01/01/2010 until 31/12/2020** | **855** |

Date of search: March 19, 2021.

**Detailed search strategy for non-interventional studies**

**MEDLINE via PubMed for non-interventional studies**

| **Search queries** | | **Hits** |
| --- | --- | --- |
| **1.** | ("Head and Neck Neoplasms"[Mesh] OR ((head OR neck OR head and neck OR oropharyngeal OR oropharynx OR pharynx OR pharyngeal OR aerodigestive tract OR oral OR oral cavity OR tonsil OR tonsillar OR tongue OR Waldeyer ring OR hypopharyngeal OR hypopharynx) AND (cancer OR neoplasm OR carcinoma))) | 515,519 |
| **2.** | ("human papillomavirus" OR HPV OR "Papillomavirus Infections"[Mesh] OR "Papillomaviridae"[Mesh]) | 66,267 |
| **3.** | "Oropharyngeal Neoplasms"[Mesh] OR "oropharyngeal cancer" OR "oropharyngeal neoplasm" OR "oropharynx neoplasm" OR "oropharynx cancer" | 10,201 |
| **4.** | ((("locoregionally advanced") OR ((locoregionally OR locoregional OR locally OR local) AND (advanced))) OR ("Neoplasm Recurrence, Local"[Mesh])) OR (("recurrent-metastatic" OR "recurrent-metastatic") OR (("Recurrence"[Mesh] OR recurrent OR recurrence OR recurren* OR relapsed OR relapse OR relaps*) AND (metastatic OR metastasis OR metasta* OR "secondary" [Subheading]))) | 338,844 |
| **5.** | #1 AND #2 | 10,928 |
| **6.** | **#5 AND #4** | **1,194** |
| **7.** | #1 AND #3 | 10,201 |
| **8.** | **#7 AND #4** | **1,641** |
| **9.** | #1 AND #2 AND #3 | 2,959 |
| **10.** | **#9 AND #4** | **431** |
| **11.** | "Epidemiology"[Mesh] OR "Epidemiology" OR "Prevalence"[Mesh] OR "Prevalence" OR "Incidence"[Mesh] OR "Incidence" OR "Disease frequency" OR "Mortality"[Mesh] OR "mortalities" OR "mortality" OR "cause of death" OR "death rate" OR "case fatality" OR "Morbidity"[Mesh] OR "morbidity" | 3,930,845 |
| **12.** | **#6 AND #11** | **580** |
| **13.** | **#8 AND #11** | **867** |
| **14.** | **#10 AND #11** | **279** |
| **15.** | "real-life data" OR "real-life evidence" OR "real-world data" OR "real-world evidence" OR "real-world use" OR "real-world effectiveness" OR "real-world" OR "real world" OR RWE OR RWD OR "cohort analysis" OR "cohort study" OR "longitudinal study" OR "observational study" OR observational OR retrospective OR prospective OR "case control study" OR "case control setting" OR "case control settings" OR "nested case control study" OR "matched case-control study" OR "case series" OR "surveillance study" OR "cross sectional" OR registry OR database OR "non-interventional studies" OR "non-interventional study" OR "non-interventional" OR "non-interventional observational study" OR "phase IV study" OR "phase 4 study" OR "phase IV trial" OR "phase 4 trial" OR "post-marketing surveillance trial" OR "post-marketing surveillance study" OR "post-authorisation study" OR "post-authorisation safety study" OR "post-authorisation efficacy study" | 3,357,526 |
| **16.** | **#6 AND #15** | **466** |
| **17.** | **#8 AND #15** | **786** |
| **18.** | **#10 AND #15** | **241** |
| **19.** | **#12 OR #16** | **752** |
| **20.** | **#13 OR #17** | **1,137** |
| **21.** | **#14 OR #18** | **346** |
| **22.** | **#19 AND Filters: from 2010 - 2020** | **640** |
| **23.** | **#20 AND Filters: from 2010 - 2020** | **660** |
| **24.** | **#21 AND Filters: from 2010 - 2020** | **324** |
| **25.** | **#19 AND Filters: from 2010 – 2020, English** | **618** |
| **26.** | **#20 AND Filters: from 2010 – 2020, English** | **635** |
| **27.** | **#21 AND Filters: from 2010 – 2020, English** | **312** |
| **28.** | **#25 OR #26 OR #27** | **941** |
| **29.** | **#25 OR #26 OR #27 AND Filters: NOT (congress[Filter] OR review[Filter] OR scientificintegrityreview[Filter] OR systematicreview[Filter])** ^†^ | **832** |

**Abbreviations**: MeSH: Medical subject heading; RWD: Real-world data; RWE: real-world evidence.

Date of search: March 19, 2021.

^†^ To exclude congress abstracts and reviews.

**EMBASE for non-interventional studies**

| **Search queries** | | **Hits** |
| --- | --- | --- |
| **1.** | 'head and neck tumor'/exp OR ((('neck'/exp OR neck OR 'head'/exp OR head) AND ('neck'/exp OR neck) OR oropharyngeal OR 'oropharynx'/exp OR oropharynx OR 'pharynx'/exp OR pharynx OR pharyngeal OR 'aerodigestive tract' OR (aerodigestive AND ('tract'/exp OR tract)) OR oral OR 'oral cavity'/exp OR 'oral cavity' OR (oral AND cavity) OR 'tonsil'/exp OR tonsil OR tonsillar OR 'tongue'/exp OR tongue OR 'waldeyer ring'/exp OR 'waldeyer ring' OR (waldeyer AND ('ring'/exp OR ring)) OR hypopharyngeal OR 'hypopharynx'/exp OR hypopharynx) AND ('cancer'/exp OR cancer OR 'neoplasm'/exp OR neoplasm OR 'carcinoma'/exp OR carcinoma)) | 750,878 |
| **2.** | 'human papillomavirus' OR hpv OR 'papillomavirus infections'/exp OR 'papillomaviridae'/exp | 96,614 |
| **3.** | 'oropharyngeal neoplasms'/exp OR 'oropharyngeal cancer' OR 'oropharyngeal neoplasm' OR 'oropharynx neoplasm' OR 'oropharynx cancer' | 15,135 |
| **4.** | 'locoregionally advanced' OR ((locoregionally OR locoregional OR locally OR local) AND advanced) OR 'neoplasm recurrence, local'/exp OR 'recurrent-metastatic' OR (('recurrence'/exp OR recurrent OR recurrence OR recurren* OR relapsed OR relapse OR relaps*) AND (metastatic OR metastasis OR metasta* OR 'secondary')) | 403,158 |
| **5.** | #1 AND #2 | 18,676 |
| **6.** | **#5 AND #4** | **2,203** |
| **7.** | #1 AND #3 | 15,135 |
| **8.** | **#7 AND #4** | **2,562** |
| **9.** | #1 AND #2 AND #3 | 5,444 |
| **10.** | **#9 AND #4** | **852** |
| **11.** | 'epidemiology'/exp OR 'epidemiology' OR 'prevalence'/exp OR 'prevalence' OR 'incidence'/exp OR 'incidence' OR 'disease frequency' OR 'mortality'/exp OR 'mortalities' OR 'mortality' OR 'cause of death' OR 'death rate' OR 'case fatality' OR 'morbidity'/exp OR 'morbidity' | 5,811,717 |
| **12.** | **#6 AND #11** | **824** |
| **13.** | **#8 AND #11** | **995** |
| **14.** | **#10 AND #11** | **392** |
| **15.** | 'real-life data' OR 'real-life evidence' OR 'real-world data' OR 'real-world evidence' OR 'real-world use' OR 'real-world effectiveness' OR 'real-world' OR 'real world' OR rwe OR rwd OR 'cohort analysis'/exp OR 'cohort analysis' OR 'cohort study'/exp OR 'cohort study' OR 'longitudinal study'/exp OR 'longitudinal study' OR 'observational study'/exp OR 'observational study' OR observational OR retrospective OR prospective OR 'case control study'/exp OR 'case control study' OR 'case control setting' OR 'case control settings' OR 'nested case control study'/exp OR 'nested case control study' OR 'matched case-control study'/exp OR 'matched case-control study' OR 'case series'/exp OR 'case series' OR 'surveillance study' OR 'cross sectional' OR 'registry'/exp OR registry OR 'database'/exp OR database OR 'non-interventional studies' OR 'non-interventional study' OR 'non-interventional' OR 'non-interventional observational study' OR 'phase iv study'/exp OR 'phase iv study' OR 'phase 4 study'/exp OR 'phase 4 study' OR 'phase iv trial'/exp OR 'phase iv trial' OR 'phase 4 trial'/exp OR 'phase 4 trial' OR 'post-marketing surveillance trial' OR 'post-marketing surveillance study' OR 'post-authorisation study' OR 'post-authorisation safety study' OR 'post-authorisation efficacy study' | 5,793,873 |
| **16.** | **#6 AND #15** | **884** |
| **17.** | **#8 AND #15** | **1,239** |
| **18.** | **#10 AND #15** | **453** |
| **19.** | **#12 OR #16** | 1341 |
| **20.** | **#13 OR #17** | 1696 |
| **21.** | **#14 OR #18** | **626** |
| **22.** | **#19 AND Filters: from 2010 - 2020** | **1233** |
| **23.** | **#20 AND Filters: from 2010 - 2020** | **1319** |
| **24.** | **#21 AND Filters: from 2010 - 2020** | **592** |
| **25.** | **#19 AND Filters: from 2010 – 2020, English** | **1211** |
| **26.** | **#20 AND Filters: from 2010 – 2020, English** | **1291** |
| **27.** | **#21 AND Filters: from 2010 – 2020, English** | **582** |
| **28.** | **#25 OR #26 OR #27** | **1920** |
| **29.** | **#25 OR #26 OR #27 AND Filters: [article]/lim OR [article in press]/lim OR [data papers]/lim OR [erratum]/lim OR [letter]/lim OR [note]/lim) AND [embase]/lim ^†^** | **931** |

Date of search: March 19, 2021.

^†^ To exclude the following: Conference Abstract, Conference Paper, Conference Review, Review, MEDLINE, and pubmed-not-MEDLINE.

## Supplementary Table S1. Characteristics of studies included in the evidence synthesis.

| **Study Acronym**  **(Source) or Primary Author (Year)** | **Study Design** | **Enrollment Period ^†^** | **Study Population ^‡^** |
| --- | --- | --- | --- |
| **INTERVENTIONAL STUDIES** | | | |
| **NCT01045421^2^**  (Full Publication) | Five-arm, non-randomized, OL, MC, phase 2 study | 2010 - 2013 | BC, SCLC, NSCLC, HNC, or gastro-oesophageal adenocarcinoma relapsed or refractory to ≤2 chemotherapy regimens not including adjuvant or neoadjuvant treatments |
| **NCT01126216^3^**  (Full Publication) | Two-arm, randomized, OL, MC, phase 3 study | 2010 - 2015 | LA SCCHN; excluding prior radiotherapy of the neck or chemotherapy and prior neck-dissection or surgical intervention exceeding an exploratory excision |
| **NCT01133678^4^**  (Full Publication) | Single-arm, non-randomized, DB, MC, phase 1/2 study | 2010 - 2014 | Treatment-naïve LA HNSCC; excluding prior chemotherapy or RT, or current immunosuppressive therapy |
| **NCT01172769^5^**  (Full Publication) | Single-arm, non-randomized, OL, MC, phase 2 study | 2010 - 2011 | Platinum- and cetuximab-refractory RM SCCHN |
| **NCT01195922^6^**  (Full Publication) | Single-arm, non-randomized, OL, MC, phase 2 study | 2010 - n/a | Previously untreated advanced HNSCC, planned for curative treatment either with surgery or CRT |
| **NCT01218048^7^**  (Full Publication) | Single-arm, non-randomized, OL, SC, phase 2 study | 2011 - n/a | Newly diagnosed, untreated, LA disease without distant metastases |
| **NCT01255800^8^**  (Full Publication) | Single-arm, non-randomized, OL, SC, phase 1 study | 2011 - n/a | RM HNSCC; prior treatment with cetuximab was allowed |
| **NCT01345682^9,10^** (Full Publication) | Two-arm, randomized, OL, MC, phase 3 study | 2012 - 2013 | RM HNSCC not amenable for salvage surgery or RT; ≥2 prior cycles of cisplatin or carboplatin |
| **NCT01379339^11^**  (Abstract) | Single-arm, non-randomized, OL, SC, phase 1 study | 2011 - n/a | Previously untreated LA SCCHN |
| **NCT01412229^12^**  (Full Publication) | Single-arm, non-randomized, OL, SC, phase 2 study | 2011 - 2015 | Previously untreated non-nasopharyngeal aerodigestive LA SCCHN |
| **NCT01417936^13^**  **(**Full Publication) | Single-arm, non-randomized, OL, MC, proof of concept phase 2 study | 2011 - 2012 | RM SCCHN not amenable to curative treatment; prior anti-EGFR mAb containing palliative treatment |
| **NCT01437449^14^**  (Full Publication) | Single-arm, non-randomized, OL, SC, phase 2 study | 2011 - n/a | Incurable RM SCCHN without prior palliative chemotherapy |
| **NCT01449201^15^**  (Full Publication) | Single-arm, non-randomized, OL, MC, phase 2 study | 2012 - 2013 | RM SCCHN; prior platinum-based chemotherapy (either cisplatin or carboplatin) ± cetuximab |
| **NCT01458392^16^**  (Full Publication) | Single-arm, non-randomized, OL, MC, phase 2 study | 2011 - 2013 | RM SCCHN; previously treated with ≥1 platinum-containing regimen or contraindicated for treatment with a platinum-containing therapy |
| **NCT01468896^17^**  (Full Publication) | Single-arm, non-randomized, OL, SC, phase 1/2 study | 2011 - n/a | Unresectable RM HNSCC; any number of  prior systemic therapies for metastatic/recurrent disease were  permitted |
| **NCT01472653^18^**  (Full Publication) | Single-arm, non-randomized, OL, SC, phase 2 study | 2011 - 2013 | Newly diagnosed, LA SCCHN, without distant metastases (M0-stage) |
| **NCT01566435^19,20^** (Full Publication) | Single-arm, non-randomized, OL, SC, phase 2 study | 2012 - 2013 | LA HNSCC; prior chemotherapy, EGFR-targeted therapy, or RT for HNSCC excluded |
| **NCT01577173^21^**  (Full Publication) | Two-arm, randomized, OL, MC, phase 2 study | 2012 - 2013 | RM SCCHN progressed after ≥1 lines of treatment, ≥1 platinum-based regimen, and not suitable for local therapy |
| **NCT01592721^22^**  (Full Publication) | Single-arm, non-randomized, OL, SC, phase 1 study | 2013 - 2014 | LA HNSCC |
| **NCT01612351^23^**  (Full Publication) | Single-arm, non-randomized, OL, SC, phase 2 study | 2012 - 2016 | Previously untreated, primary LA SCCHN |
| **NCT01696955^24,25^** (Full Publication & Abstract) | Two-arm, randomized, OL, MC, phase 2 study | 2012 - 2014 | RM HNSCC; not amenable to curative-intent therapy |
| **NCT01716416^26^**  (Full Publication) | Single-arm, non-randomized, OL, SC, phase 1b/2 study | 2013 - 2017 | RM HNSCC; prior EGFR-targeted therapy for RM disease excluded |
| **NCT01737008^27^** (Full Publication) | Single-arm, non-randomized, OL, SC, phase 1b/2 study | 2013 - n/a | LA SCCHN; prior exposure to biological agents targeting EGFR excluded |
| **NCT01816984^28^**  (Full Publication) | Single-arm, non-randomized, OL, SC, phase 1b study | 2013 - 2015 | RM HNSCC; not amenable to curative intent therapy; no prior treatment with a PI3K inhibitor or ≥2 prior lines of cytotoxic chemotherapy for RM disease |
| **NCT01836029^29^**  **(**Full Publication) | Two-arm, randomized, DB, MC, phase 2 study | 2013 - 2015 | RM SCCHN; no previous systemic treatment for RM disease; not amenable to curative local therapy |
| **NCT01848834^30-32^** (Full Publication) | Single-arm, non-randomized, OL, MC, phase 1b study | 2013 - 2014 | RM SCCHN (initial cohort 60 PDL1+ patients and expanded cohort with 132 patients regardless of PDL1 status); no limit to number of prior therapies; prior treatment with ICIs excluded |
| **NCT01856478^33^**  (Full Publication) | Two-arm, randomized, OL, MC, phase 3 trial | 2013 - 2018 | RM SCCHN; after 1 platinum-based chemotherapy; not amenable to salvage surgery or RT; primary tumor site of nasopharynx (of any histology), sinuses, and/or salivary glands excluded; prior EGFR-targeted therapy excluded; |
| **NCT01911598^34^**  (Full Publication) | Single-arm, non-randomized, OL, MC, phase 1b study | 2013 - 2014 | RM SCCHN; not amenable to further curative local therapy; no prior chemotherapy |
| **NCT01935921^35^**  (Abstract) | Single-arm, non-randomized, OL, SC, phase 1 study | 2013 - 2016 | Previously untreated LA HNSCC HR (HPV‐) or IR (HPV+ and either: ≥ 10 pack-year tobacco and ≥ N2 disease; or T4 or N3 disease) |
| **NCT01946867^36^**  (Full Publication) | Single-arm, non-randomized, OL, MC, phase 1 study | 2014 - 2018 | Elderly patients (>65years) with LA SCCHN; prior RT excluded |
| **NCT01969877^37^** (Full Publication) | Two-arm, randomized, OL, MC, phase 3 study | 2013 - 2018 | LA HNSCC without distant metastases |
| **NCT02052960^38,39^** (Abstract) | Two-arm, randomized, OL, MC, phase 2 study | 2014 - n/a | RM HNSCC; prior systemic chemotherapy, cetuximab, and EGFR-targeted therapy excluded |
| **NCT02105636^40^** (Full Publication) | Two-arm, randomized, OL, MC, phase 3 study | 2014 - 2015 | Platinum-refractory RM SCCHN not amenable to curative treatment |
| **NCT02207530^41^** (Full Publication) | Single-arm, non-randomized, OL, MC, phase 2 study | 2014 - 2017 | RM HNSCC not amenable to therapy with curative intent and with PDL1-high (TC≥25%) expression; ≤1 prior platinum-based regimen for RM disease |
| **NCT02252042^42^** (Full Publication) | Two-arm, randomized, OL, MC, phase 3 study | 2014 - 2016 | RM HNSCC; prior platinum-containing regimen; ≤2 prior lines of therapy for RM disease |
| **NCT02255097^43^** (Full Publication) | Single-arm, non-randomized, OL, MC, phase 2 study | 2014 - 2015 | Platinum- and cetuximab-resistant RM HNSCC |
| **NCT02268695^44^** (Full Publication) | Two-arm, randomized, OL, MC, phase 2 study | 2014 - 2017 | RM HNSCC unsuitable for curative treatment; aged 18-70; patients with nasopharyngeal cancer, paranasal sinus cancer or unknown primary were excluded |
| **NCT02274155^45^** (Full Publication) | Single-arm, non-randomized, OL, SC, phase 1 study | 2014 –2017 | LA HNSCC considered surgically resectable |
| **NCT02277197^46,47^**  (Full Publication) | Single-arm, non-randomized, OL, MC, phase 1b study | 2015 – 2016 | Cetuximab-resistant RM HNSCC |
| **NCT02282371^48^** (Full Publication) | Single-arm, non-randomized, OL, MC, phase 1b study | 2015 – 2017 | LA HNSCC; nasopharyngeal cancer was excluded |
| **NCT02308072^49^** (Full Publication) | Single-arm, non-randomized, OL, MC, phase 1 study | 2012 - 2016 | Heavy smoker (≥10 pack-years) patients with LA HNC |
| **NCT02319044^50^** (Full Publication) | Three-arm, randomized, OL, MC, phase 2 study | 2015 - 2016 | RM HNSCC; ≤1 platinum-containing regimen for RM disease; non-squamous histologies (eg, nasopharynx or salivary gland) excluded |
| **NCT02350712^51^** (Full Publication) | Single-arm, non-randomized, OL, MC, phase 1 study | 2014 - 2015 | RM SCCHN; prior anti-EGFR/HER2/HER3/HER4 targeted therapy and prior treatment for RM disease excluded |
| **NCT02358031^52^** (Full Publication) | Three-arm, randomized, OL, MC, phase 3 study | 2015 - 2017 | Locally incurable RM HNSCC and no prior systemic therapy in the RM setting |
| **NCT02369874^53^** (Full Publication) | Three-arm, randomized, OL, MC, phase 3 study | 2015 - 2017 | RM HNSCC not amenable to curative therapy; ≤1 prior platinum-containing regimen |
| **NCT02508389^54^** (Full Publication) | Two-arm, randomized, DB, MC, phase 2b study | 2015 - n/a | Patients undergoing CRT for LA Non-Metastatic SCC of the Oral Cavity/Oropharynx; patients with oral mucositis (WHO Score ≥ Grade 1) at study entry were excluded |
| **NCT02537223^55^** (Full Publication) | Single-arm, non-randomized, OL, SC, phase 1 study | 2015 - 2017 | Previously untreated LA SCCHN; candidates for CRT; tumors arising from the nasopharynx, skin or unknown site excluded |
| **NCT02538510^56^** (Full Publication) | Single-arm, non-randomized, OL, SC, phase 1/2 study | 2015 - 2017 | Progressing incurable HNC and SGC |
| **NCT02549742^57^** (Full Publication) | Single-arm, non-randomized, OL, SC, phase 2 study | 2014 - 2017 | Patients with recurrent mucosal HNC with no curative treatment options |
| **NCT02573493^58^** (Full Publication) | Two-arm, non-randomized, OL, MC, phase 2 study | 2016 – 2019 | LA HNSCC |
| **NCT02586207^59^** (Full Publication) | Single-arm, non-randomized, OL, MC, phase 1b study | 2015 - 2018 | LA HNSCC eligible for cisplatin-based, standard-dose (70 Gy) CRT |
| **NCT02609503^60^** (Full Publication) | Single-arm, non-randomized, OL, MC, phase 2 study | 2016 - 2018 | Cisplatin ineligible patients with untreated LA HNSCC; nasopharyngeal carcinoma excluded |
| **NCT02626000^61^** (Full Publication) | Single-arm, non-randomized, OL, MC, phase 1b study | 2016 - 2017 | RM HNSCC refractory to platinum-based chemotherapy |
| **NCT02643056^62^** (Full Publication) | Single-arm, non-randomized, OL, MC, phase 2 study | 2011 - n/a | Platinum-pretreated HNC |
| **NCT02707588^63,64^** (Abstract) | Two-arm, randomized, OL, SC, phase 2 study | 2017 | Patients unfit for high dose cisplatin, non-operable LA SCC |
| **NCT02718820^65^** (Abstract) | Single-arm, non-randomized, OL, SC, phase 1/2 study | 2016 - n/a | RM HNSCC; failure of prior platinum therapy |
| **NCT02764593**^66^ (Abstract) | Four-arm, OL, non-randomized, MC phase 1 study | 2016 - n/a | Newly diagnosed IR/HR LA HNSCC |
| **NCT02938273^67^** (Full Publication) | Single-arm, non-randomized, OL, SC, phase 1 study | 2017 - 2018 | Advanced-stage HNSCC; naïve for immunotherapy |
| **NCT02999087^68^** (Full Publication) | Four-arm, OL, randomized, MC phase 3 study | 2017 - 2018 | Previously untreated LA SCCHN |
| **NCT03003637^69^** (Abstract) | Single-arm, non-randomized, OL, SC, phase 1/2 study | 2017 – n/a | Advanced or recurrent HNSCC indicated for curative (salvage) surgery |
| **NCT03370276^70^** (Full Publication) | Single-arm, non-randomized, OL, MC, phase 1/2 study | 2017 –2019 | RM SCCHN; prior failure of ≥1 line of palliative treatment for incurable HNSCC |
| **NCT04397341^71^** (Full Publication) | Single-arm, non-randomized, OL, SC, phase 2 study | 2014 - 2015 | Unresectable LA SCCHN; previous chemotherapy or RT for SCCHN excluded |
| **NON-INTERVENTIONAL STUDIES** | | | |
| **Bossi (2016)^72^** | Prospective cohort, SC | 2011 –2012 | Patients with LA HNSCC requiring CRT; patients with oral mucositis at baseline were excluded |
| **Bossi (2019)^73^** | Cross-sectional, SC | 2015 –2016 | Patients with LA HNSCC having completed curative treatment at least 1 year earlier and free of disease; ≥18 years old |
| **Botticelli (2020)^74^** | Retrospective/ prospective, MC | 2018 –2020 | Patients with platinum refractory RM HNSCC who received nivolumab 240 mg every 2 weeks |
| **Byrne (2019)^75^** | Cross-sectional/ retrospective, MC | 2016 –2016 | Patients with advanced (LA and RM) HNSCC; receiving active drug treatment; ≥18 years old |
| **Castelli (2019)^76^** | Retrospective cohort, MC | 2010 –2017 | Patients with LA HNC treated with definitive concurrent CRT or RT and cetuximab; ≥18 – 75 years old |
| **de Ridder (2020)^77^** | Retrospective cohort, SC | 2010 – 2017 | Patients with recurrent HNSCC after CRT in the time period after the first recurrence |
| **Galot (2020)^78^** | Cross-sectional, SC | 2013 – n/a | Patients with incurable locoregional recurrent and/or metastatic HNSCC treated with standard of care at the institution; ≥18 years old |
| **Grünwald (2020)^79^** | Retrospective cohort, MC | 2011 – 2013 | Patients who initiated first-line systemic therapy for RM HNSCC; adult patients |
| **Hilke (2020)^80^** | Prospective pilot study, SC | 2015 – 2016 | Patients with LA HNSCC receiving definitive CRT after primary diagnosis |
| **Kim (2020)^81^** | Retrospective cohort, SC | 2016 –2019 | Patients with RM HNC, previously treated with chemotherapy, RT or CRT, and who received pembrolizumab or nivolumab (oropharynx and oral cavity only) |
| **Martens (2019)^82^** | Retrospective cohort, n/a | 2012 – 2017 | Patients with HNSCC with or without lymph node metastasis; pretreatment DWI and/or 18F-FDG PET/CT and planned CRT; previous locoregional treatment excluded |
| **Martens (2020)^83^** | Retrospective cohort, SC | 2012 – 2018 | Patients with previously untreated LA HNSCC, planned for CRT; ≥18 years old |
| **Nadler (2019)^84^** | Retrospective cohort, SC | 2011 –2014 | Patients with RM HNSCC who initiated systemic therapy; ≥18 years old |
| **Noij (2018)^85^** | Retrospective cohort, SC | 2012 –2015 | Patients with advanced stage HNSCC with both DWI and 18F-FDG PET/CT 3-6 months after CRT |
| **Pitak-Arnnop (2020)^86^** | Retrospective cohort, SC | 2015 –2017 | Patients with SCCPOCO treated at the institution |
| **Porter (2020)^87^** | Retrospective cohort, SC | 2015 –2016 | Patients with RM HNC who received care at the institution |
| **Smirk (2018)^88^** | Retrospective cohort, SC | 2014 –2017 | Patients who had salvage procedures for recurrent oral and oropharyngeal carcinoma |
| **Sridharan (2018)^89^** | Retrospective cohort, SC | 2014 – 2017 | Patients with advanced HNSCC treated with single-agent ICPi using a PD-1 inhibitor |
| **Velez (2018)^90^** | Retrospective cohort, MC | 2011 –2016 | Patients who underwent re-irradiation for HNSCC occurring in a previously irradiated field; patients with biopsy-proven recurrent disease |

**Abbreviations**: 18F-FDG PET/CT, 18F-Fluorodeoxyglucose Positron Emission Tomography Combined With Computed Tomography; BC, breast cancer; CRT, chemoradiation; DB, double-blind; DWI, diffusion-weighted imaging; EGFR, epidermal growth factor receptor; Gy, Grey; HER2/HER3/HER4, human epidermal growth factor receptor 2/3/4; HNC, head and neck cancer; HNSCC, head and neck squamous cell carcinoma; HPV, human papillomavirus; HR, high risk; ICI, immune checkpoint inhibitors; ICPi, immune checkpoint inhibitor; IR, intermediate risk; LA, locally and regionally advanced; M, metastasis; mAb, mouse antibody; MC, multicenter; N, node; n/a, not available; NSCLC, non-small cell lung cancer; OL, open-label; PD-1, programmed cell death protein -1; PDL1, programmed death-ligand-1; PI3K, phosphatidylinositol-3-kinase; RM, recurrent and/or metastatic; RT, radiotherapy; SC, single center; SCC, squamous cell carcinoma; SCCHN, squamous cell carcinoma of the head and neck; SCCPOCO, squamous cell carcinoma of the posterior oral cavity and oropharynx; SCLC, small cell lung cancer; SGC, salivary gland cancer; T, tumor; TC, tumor cell; WHO, world health organization.

† If enrollment period was not available from the publication or abstract, study start date was retrieved from clinicaltrials.gov/NCT#; ‡ For non-interventional studies, eligible age range is also provided, if specified in the publication.

## Supplementary Figure S1. Summary of HPV prevalence in LA and RM (A) HNC and (B) OPC per geographic region.


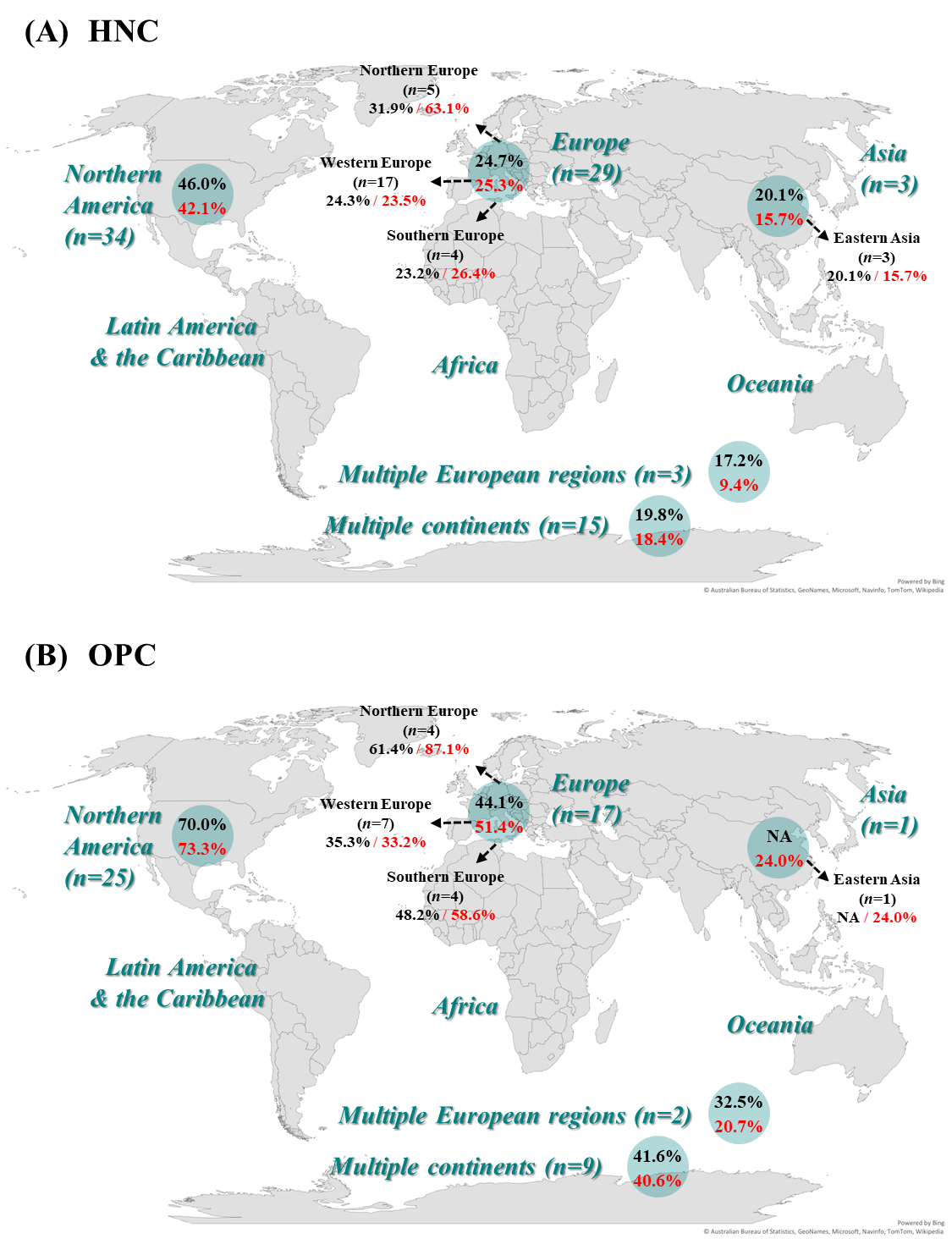


HNC, head and neck cancer; HPV, human papilloma virus; *n*, number of studies; NA, not applicable; OPC, oropharyngeal cancer.

HPV prevalence per region is provided as mean and pooled HPV prevalence across studies shown in black and red, respectively. The category of Multiple European regions includes multi-country studies conducted in Europe; these studies were conducted in Western, Central/Eastern, and Southern Europe for HNC, and Southern and Western Europe for OPC.

## Supplementary Figure S2. HPV prevalence in LA and RM (A) HNC and (B) OPC per region and disease stage.


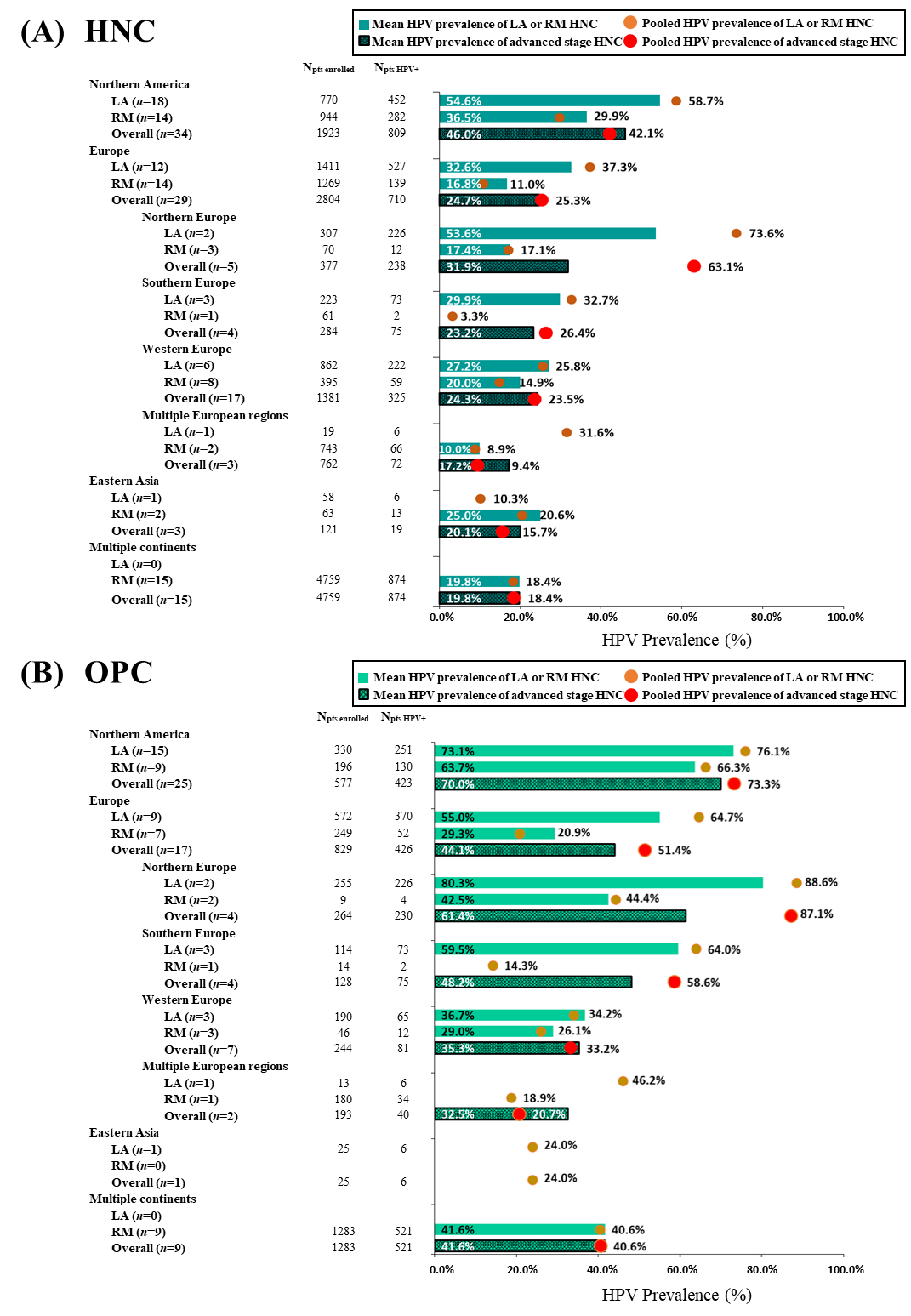


HPV, human papilloma virus; LA, locally and regionally advanced; N, number of patients; *n*, number of studies; OPC, oropharyngeal cancer; RM, recurrent and/or metastatic.

HPV prevalence is provided as mean (bar) and pooled (circle) HPV prevalence across studies in each subgroup. LA and/or RM HNC includes LA, RM, as well as advanced stage as defined by the author or both LA and RM.

## Supplementary Figure S3. HPV detection methodologies, (A) overall and (B) by study design, disease stage and anatomical site of assessment.


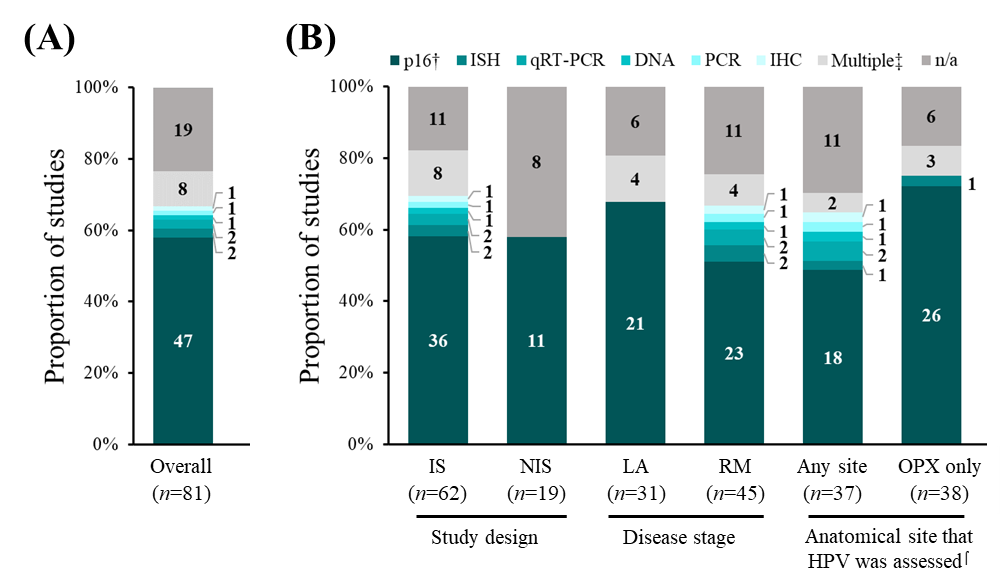


DNA, deoxyribonucleic acid; HNC, head and neck cancer; HPV, human papilloma virus; IHC, immunohistochemistry; ISH, in situ hybridization; LA, locally and regionally advanced; *n*, number of studies; n/a, not available; OPX, oropharynx; p16, p16^INK4A^ protein; PCR, polymerase chain reaction; qRT-PCR, quantitative reverse transcription PCR; RM, recurrent and/or metastatic.

The distribution of studies per HPV detection methodology is shown overall (A) and in each subgroup (B). Numbers inside bars indicate number of studies. † Including studies where initial screening for HPV was performed by p16 detection and further confirmation of positivity was performed using other methods as well. ‡ Multiple refers to the number of assays reported in each study without necessarily referring to a combined approach per subject. ⌠ Studies were grouped by anatomical site that HPV was assessed into “Any site” meaning that any head and neck sub-sites enrolled, including OPX, were tested for HPV status, and “OPX only” representing studies where HPV status was tested only in OPX, in which case HPV positivity is available from OPC patients only even though patients with HNC in other head and neck sub-sites had also been enrolled in the study.

## Supplementary Figure S4. HPV prevalence in advanced OPC tested for HPV positivity using solely a p16-based assay


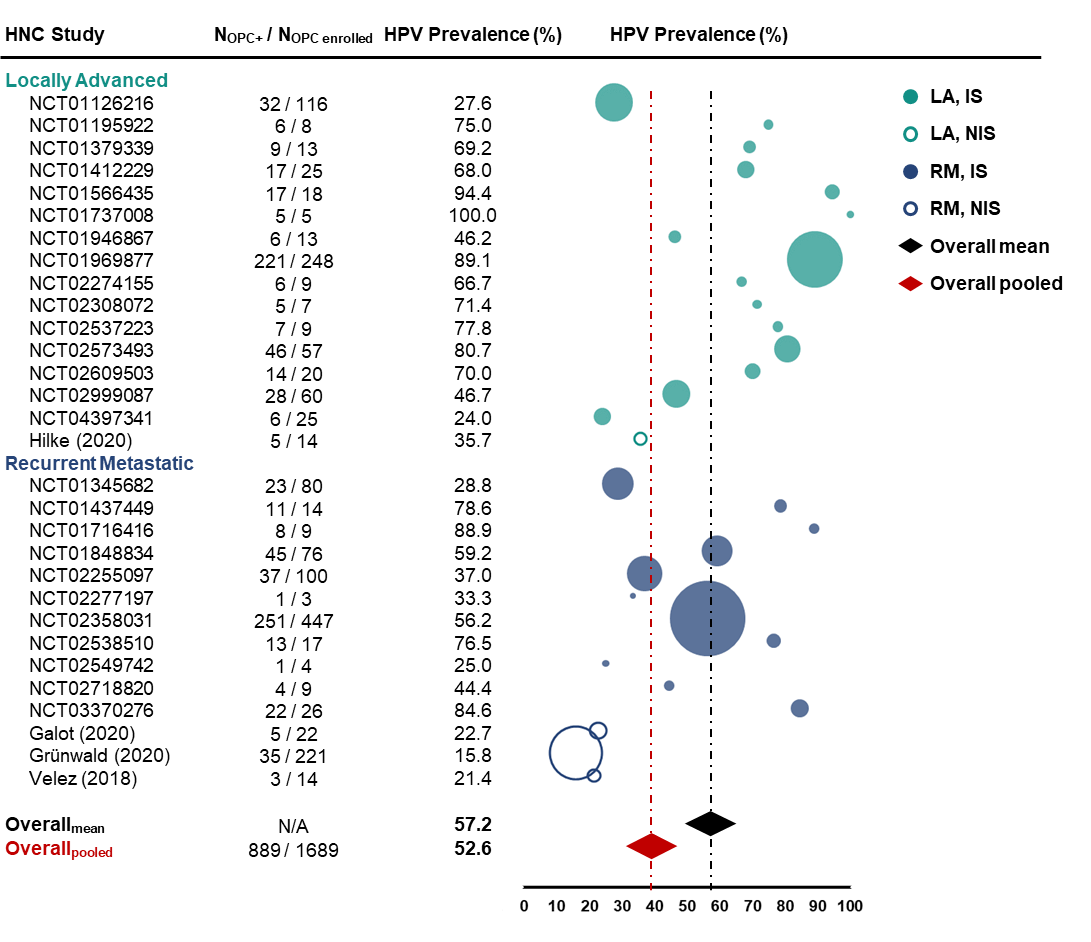


HNC, head and neck cancer; HPV, human papilloma virus; IS, interventional studies; LA, locally and regionally advanced; N, number of patients; N/A, not applicable; NIS, non-interventional studies; OPC, oropharyngeal cancer; p16, p16^INK4A^ protein; RM, recurrent and/or metastatic.

Circle size corresponds to number of patients included in the study indicated, ranging from 3 to 447 patients across 30 studies in OPC. Overall HPV prevalence is provided as mean and pooled HPV prevalence across studies and depicted as a black and red diamond, respectively.

# References

1. Page MJ, McKenzie JE, Bossuyt PM, Boutron I, Hoffmann TC, Mulrow CD, et al. The PRISMA 2020 statement: an updated guideline for reporting systematic reviews. BMJ. 2021;372:n71. <https://doi.org/10.1136/bmj.n71>.

2. Melichar B, Adenis A, Lockhart AC, Bennouna J, Dees EC, Kayaleh O, et al. Safety and activity of alisertib, an investigational aurora kinase A inhibitor, in patients with breast cancer, small-cell lung cancer, non-small-cell lung cancer, head and neck squamous-cell carcinoma, and gastro-oesophageal adenocarcinoma: a five-arm phase 2 study. The Lancet Oncology. 2015;16(4):395-405. <https://doi.org/10.1016/s1470-2045(15)70051-3>.

3. Fietkau R, Hecht M, Hofner B, Lubgan D, Iro H, Gefeller O, et al. Randomized phase-III-trial of concurrent chemoradiation for locally advanced head and neck cancer comparing dose reduced radiotherapy with paclitaxel/cisplatin to standard radiotherapy with fluorouracil/cisplatin: The PacCis-trial. Radiotherapy and oncology : journal of the European Society for Therapeutic Radiology and Oncology. 2020;144:209-17. <https://doi.org/10.1016/j.radonc.2020.01.016>.

4. Villaflor VM, Melotek JM, Karrison TG, Brisson RJ, Blair EA, Portugal L, et al. Response-adapted volume de-escalation (RAVD) in locally advanced head and neck cancer. Annals of oncology : official journal of the European Society for Medical Oncology. 2016;27(5):908-13. <https://doi.org/10.1093/annonc/mdw051>.

5. Grünwald V, Keilholz U, Boehm A, Guntinas-Lichius O, Hennemann B, Schmoll HJ, et al. TEMHEAD: a single-arm multicentre phase II study of temsirolimus in platin- and cetuximab refractory recurrent and/or metastatic squamous cell carcinoma of the head and neck (SCCHN) of the German SCCHN Group (AIO). Annals of oncology : official journal of the European Society for Medical Oncology. 2015;26(3):561-7. <https://doi.org/10.1093/annonc/mdu571>.

6. Day TA, Shirai K, O'Brien PE, Matheus MG, Godwin K, Sood AJ, et al. Inhibition of mTOR Signaling and Clinical Activity of Rapamycin in Head and Neck Cancer in a Window of Opportunity Trial. Clinical cancer research : an official journal of the American Association for Cancer Research. 2019;25(4):1156-64. <https://doi.org/10.1158/1078-0432.ccr-18-2024>.

7. Li J, Srivastava RM, Ettyreddy A, Ferris RL. Cetuximab ameliorates suppressive phenotypes of myeloid antigen presenting cells in head and neck cancer patients. Journal for immunotherapy of cancer. 2015;3:54. <https://doi.org/10.1186/s40425-015-0097-6>.

8. Bowles DW, Keysar SB, Eagles JR, Wang G, Glogowska MJ, McDermott JD, et al. A pilot study of cetuximab and the hedgehog inhibitor IPI-926 in recurrent/metastatic head and neck squamous cell carcinoma. Oral Oncol. 2016;53:74-9. <https://doi.org/10.1016/j.oraloncology.2015.11.014>.

9. Machiels JP, Haddad RI, Fayette J, Licitra LF, Tahara M, Vermorken JB, et al. Afatinib versus methotrexate as second-line treatment in patients with recurrent or metastatic squamous-cell carcinoma of the head and neck progressing on or after platinum-based therapy (LUX-Head & Neck 1): an open-label, randomised phase 3 trial. The Lancet Oncology. 2015;16(5):583-94. <https://doi.org/10.1016/s1470-2045(15)70124-5>.

10. Cohen EEW, Licitra LF, Burtness B, Fayette J, Gauler T, Clement PM, et al. Biomarkers predict enhanced clinical outcomes with afatinib versus methotrexate in patients with second-line recurrent and/or metastatic head and neck cancer. Annals of oncology : official journal of the European Society for Medical Oncology. 2017;28(10):2526-32. <https://doi.org/10.1093/annonc/mdx344>.

11. Camille N, Babu R, Bakst RL, Gupta V, Miles B, Genden EM, et al. Phase I study of cabazitaxel-PF induction chemotherapy in patients with locally advanced squamous cell carcinoma of the head and neck (SCCHN). Journal of Clinical Oncology. 2014;32(15_suppl):e17009. <https://doi.org/10.1200/jco.2014.32.15_suppl.e17009>.

12. Weiss J, Gilbert J, Deal AM, Weissler M, Hilliard C, Chera B, et al. Induction chemotherapy with carboplatin, nab-paclitaxel and cetuximab for at least N2b nodal status or surgically unresectable squamous cell carcinoma of the head and neck. Oral Oncol. 2018;84:46-51. <https://doi.org/10.1016/j.oraloncology.2018.06.028>.

13. Machiels JP, Specenier P, Krauß J, Dietz A, Kaminsky MC, Lalami Y, et al. A proof of concept trial of the anti-EGFR antibody mixture Sym004 in patients with squamous cell carcinoma of the head and neck. Cancer chemotherapy and pharmacology. 2015;76(1):13-20. <https://doi.org/10.1007/s00280-015-2761-4>.

14. Trieu V, Pinto H, Riess JW, Lira R, Luciano R, Coty J, et al. Weekly Docetaxel, Cisplatin, and Cetuximab in Palliative Treatment of Patients with Squamous Cell Carcinoma of the Head and Neck. The oncologist. 2018;23(7):764-e86. <https://doi.org/10.1634/theoncologist.2017-0618>.

15. Kim HS, Kwon HJ, Jung I, Yun MR, Ahn MJ, Kang BW, et al. Phase II clinical and exploratory biomarker study of dacomitinib in patients with recurrent and/or metastatic squamous cell carcinoma of head and neck. Clinical cancer research : an official journal of the American Association for Cancer Research. 2015;21(3):544-52. <https://doi.org/10.1158/1078-0432.ccr-14-1756>.

16. Jimeno A, Posner MR, Wirth LJ, Saba NF, Cohen RB, Popa EC, et al. A phase 2 study of dalantercept, an activin receptor-like kinase-1 ligand trap, in patients with recurrent or metastatic squamous cell carcinoma of the head and neck. Cancer. 2016;122(23):3641-9. <https://doi.org/10.1002/cncr.30317>.

17. McMichael EL, Benner B, Atwal LS, Courtney NB, Mo X, Davis ME, et al. A Phase I/II Trial of Cetuximab in Combination with Interleukin-12 Administered to Patients with Unresectable Primary or Recurrent Head and Neck Squamous Cell Carcinoma. Clinical cancer research : an official journal of the American Association for Cancer Research. 2019;25(16):4955-65. <https://doi.org/10.1158/1078-0432.ccr-18-2108>.

18. Strojan P, Zakotnik B, Žumer B, Karner K, Dremelj M, Jančar B, et al. Skin Reaction to Cetuximab as a Criterion for Treatment Selection in Head and Neck Cancer. Anticancer research. 2018;38(7):4213-20. <https://doi.org/10.21873/anticanres.12717>.

19. Adkins D, Ley J, Michel L, Wildes TM, Thorstad W, Gay HA, et al. nab-Paclitaxel, cisplatin, and 5-fluorouracil followed by concurrent cisplatin and radiation for head and neck squamous cell carcinoma. Oral oncology. 2016;61:1-7. <https://doi.org/10.1016/j.oraloncology.2016.07.015>.

20. Adkins D, Ley J, Oppelt P, Gay HA, Daly M, Paniello RC, et al. Impact on Health-Related Quality of Life of Induction Chemotherapy Compared With Concurrent Cisplatin and Radiation Therapy in Patients With Head and Neck Cancer. Clinical oncology (Royal College of Radiologists (Great Britain)). 2019;31(9):e123-e31. <https://doi.org/10.1016/j.clon.2019.05.007>.

21. Fayette J, Wirth L, Oprean C, Udrea A, Jimeno A, Rischin D, et al. Randomized Phase II Study of Duligotuzumab (MEHD7945A) vs. Cetuximab in Squamous Cell Carcinoma of the Head and Neck (MEHGAN Study). Frontiers in oncology. 2016;6:232. <https://doi.org/10.3389/fonc.2016.00232>.

22. Bauman JE, Duvvuri U, Thomas S, Gooding WE, Clump DA, Karlovits B, et al. Phase 1 study of EGFR-antisense DNA, cetuximab, and radiotherapy in head and neck cancer with preclinical correlatives. Cancer. 2018;124(19):3881-9. <https://doi.org/10.1002/cncr.31651>.

23. Weiss JM, Grilley-Olson JE, Deal AM, Zevallos JP, Chera BS, Paul J, et al. Phase 2 trial of neoadjuvant chemotherapy and transoral endoscopic surgery with risk-adapted adjuvant therapy for squamous cell carcinoma of the head and neck. Cancer. 2018;124(14):2986-92. <https://doi.org/10.1002/cncr.31526>.

24. Kochanny SE, Worden FP, Adkins DR, Lim DW, Bauman JE, Wagner SA, et al. A randomized phase 2 network trial of tivantinib plus cetuximab versus cetuximab in patients with recurrent/metastatic head and neck squamous cell carcinoma. Cancer. 2020;126(10):2146-52. <https://doi.org/10.1002/cncr.32762>.

25. Vokes EE, Worden FP, Adkins D, Bauman JE, Lim D, Sukari A, et al. A randomized phase II trial of the MET inhibitor tivantinib + cetuximab versus cetuximab alone in patients with recurrent/metastatic head and neck cancer. Journal of Clinical Oncology. 2015;33(15_suppl):6060-. <https://doi.org/10.1200/jco.2015.33.15_suppl.6060>.

26. Adkins D, Mehan P, Ley J, Siegel MJ, Siegel BA, Dehdashti F, et al. Pazopanib plus cetuximab in recurrent or metastatic head and neck squamous cell carcinoma: an open-label, phase 1b and expansion study. The Lancet Oncology. 2018;19(8):1082-93. <https://doi.org/10.1016/s1470-2045(18)30350-4>.

27. Chiu JW, Chan K, Chen EX, Siu LL, Abdul Razak AR. Pharmacokinetic assessment of dacomitinib (pan-HER tyrosine kinase inhibitor) in patients with locally advanced head and neck squamous cell carcinoma (LA SCCHN) following administration through a gastrostomy feeding tube (GT). Investigational new drugs. 2015;33(4):895-900. <https://doi.org/10.1007/s10637-015-0245-3>.

28. Brisson RJ, Kochanny S, Arshad S, Dekker A, DeSouza JA, Saloura V, et al. A pilot study of the pan-class I PI3K inhibitor buparlisib in combination with cetuximab in patients with recurrent or metastatic head and neck cancer. Head & neck. 2019;41(11):3842-9. <https://doi.org/10.1002/hed.25910>.

29. Ferris RL, Saba NF, Gitlitz BJ, Haddad R, Sukari A, Neupane P, et al. Effect of Adding Motolimod to Standard Combination Chemotherapy and Cetuximab Treatment of Patients With Squamous Cell Carcinoma of the Head and Neck: The Active8 Randomized Clinical Trial. JAMA oncology. 2018;4(11):1583-8. <https://doi.org/10.1001/jamaoncol.2018.1888>.

30. Chow LQM, Haddad R, Gupta S, Mahipal A, Mehra R, Tahara M, et al. Antitumor Activity of Pembrolizumab in Biomarker-Unselected Patients With Recurrent and/or Metastatic Head and Neck Squamous Cell Carcinoma: Results From the Phase Ib KEYNOTE-012 Expansion Cohort. Journal of Clinical Oncology. 2016;34(32):3838-45. <https://doi.org/10.1200/jco.2016.68.1478>.

31. Seiwert TY, Burtness B, Mehra R, Weiss J, Berger R, Eder JP, et al. Safety and clinical activity of pembrolizumab for treatment of recurrent or metastatic squamous cell carcinoma of the head and neck (KEYNOTE-012): an open-label, multicentre, phase 1b trial. The Lancet Oncology. 2016;17(7):956-65. <https://doi.org/10.1016/s1470-2045(16)30066-3>.

32. Mehra R, Seiwert TY, Gupta S, Weiss J, Gluck I, Eder JP, et al. Efficacy and safety of pembrolizumab in recurrent/metastatic head and neck squamous cell carcinoma: pooled analyses after long-term follow-up in KEYNOTE-012. British journal of cancer. 2018;119(2):153-9. <https://doi.org/10.1038/s41416-018-0131-9>.

33. Guo Y, Ahn MJ, Chan A, Wang CH, Kang JH, Kim SB, et al. Afatinib versus methotrexate as second-line treatment in Asian patients with recurrent or metastatic squamous cell carcinoma of the head and neck progressing on or after platinum-based therapy (LUX-Head & Neck 3): an open-label, randomised phase III trial. Annals of oncology : official journal of the European Society for Medical Oncology. 2019;30(11):1831-9. <https://doi.org/10.1093/annonc/mdz388>.

34. Jimeno A, Machiels JP, Wirth L, Specenier P, Seiwert TY, Mardjuadi F, et al. Phase Ib study of duligotuzumab (MEHD7945A) plus cisplatin/5-fluorouracil or carboplatin/paclitaxel for first-line treatment of recurrent/metastatic squamous cell carcinoma of the head and neck. Cancer. 2016;122(24):3803-11. <https://doi.org/10.1002/cncr.30256>.

35. Ferris RL, Clump DA, Ohr J, Gooding W, Kim S, Karlovits BJ, et al. 1139 - Phase I trial of cetuximab, intensity modulated radiotherapy (IMRT), and ipilimumab in previously untreated, locally advanced head and neck squamous cell carcinoma (PULA HNSCC). Annals of Oncology. 2017;28(suppl_5):v372-v94. <https://doi.org/10.1093/annonc/mdx374.014>.

36. Hoffmann C, Calugaru V, Borcoman E, Moreno V, Calvo E, Liem X, et al. Phase I dose-escalation study of NBTXR3 activated by intensity-modulated radiation therapy in elderly patients with locally advanced squamous cell carcinoma of the oral cavity or oropharynx. European journal of cancer (Oxford, England : 1990). 2021;146:135-44. <https://doi.org/10.1016/j.ejca.2021.01.007>.

37. Gebre-Medhin M, Brun E, Engström P, Haugen Cange H, Hammarstedt-Nordenvall L, Reizenstein J, et al. ARTSCAN III: A Randomized Phase III Study Comparing Chemoradiotherapy With Cisplatin Versus Cetuximab in Patients With Locoregionally Advanced Head and Neck Squamous Cell Cancer. Journal of Clinical Oncology. 2020;39(1):38-47. <https://doi.org/10.1200/JCO.20.02072>.

38. Baumeister H, Zurlo A, Fayette J, Dietrich B, Keilholz U. CetuGEX and cetuximab in recurrent/metastatic squamous cell carcinoma of the head and neck (RM-HNSCC): PK/PD results from the phase II RESGEX study. Journal of Clinical Oncology. 2018;36(5_suppl):61-. <https://doi.org/10.1200/JCO.2018.36.5_suppl.61>.

39. Keilholz U, Kawecki A, Dietz A, Zurawski B, Schenker M, Kukielka-Budny B, et al. Efficacy and safety of CetuGEX in recurrent/metastatic squamous cell carcinoma of the head and neck (RM-HNSCC): Results from the randomized phase II RESGEX study. Journal of Clinical Oncology. 2018;36(5_suppl):59-. <https://doi.org/10.1200/JCO.2018.36.5_suppl.59>.

40. Ferris RL, Blumenschein G, Jr., Fayette J, Guigay J, Colevas AD, Licitra L, et al. Nivolumab vs investigator's choice in recurrent or metastatic squamous cell carcinoma of the head and neck: 2-year long-term survival update of CheckMate 141 with analyses by tumor PD-L1 expression. Oral Oncol. 2018;81:45-51. <https://doi.org/10.1016/j.oraloncology.2018.04.008>.

41. Zandberg DP, Algazi AP, Jimeno A, Good JS, Fayette J, Bouganim N, et al. Durvalumab for recurrent or metastatic head and neck squamous cell carcinoma: Results from a single-arm, phase II study in patients with ≥25% tumour cell PD-L1 expression who have progressed on platinum-based chemotherapy. European journal of cancer (Oxford, England : 1990). 2019;107:142-52. <https://doi.org/10.1016/j.ejca.2018.11.015>.

42. Cohen EEW, Soulières D, Le Tourneau C, Dinis J, Licitra L, Ahn MJ, et al. Pembrolizumab versus methotrexate, docetaxel, or cetuximab for recurrent or metastatic head-and-neck squamous cell carcinoma (KEYNOTE-040): a randomised, open-label, phase 3 study. Lancet (London, England). 2019;393(10167):156-67. <https://doi.org/10.1016/s0140-6736(18)31999-8>.

43. Bauml J, Seiwert TY, Pfister DG, Worden F, Liu SV, Gilbert J, et al. Pembrolizumab for Platinum- and Cetuximab-Refractory Head and Neck Cancer: Results From a Single-Arm, Phase II Study. J Clin Oncol. 2017;35(14):1542-9. <https://doi.org/10.1200/jco.2016.70.1524>.

44. Guigay J, Aupérin A, Fayette J, Saada-Bouzid E, Lafond C, Taberna M, et al. Cetuximab, docetaxel, and cisplatin versus platinum, fluorouracil, and cetuximab as first-line treatment in patients with recurrent or metastatic head and neck squamous-cell carcinoma (GORTEC 2014-01 TPExtreme): a multicentre, open-label, randomised, phase 2 trial. The Lancet Oncology. 2021;22(4):463-75. <https://doi.org/10.1016/s1470-2045(20)30755-5>.

45. Duhen R, Ballesteros-Merino C, Frye AK, Tran E, Rajamanickam V, Chang S-C, et al. Neoadjuvant anti-OX40 (MEDI6469) therapy in patients with head and neck squamous cell carcinoma activates and expands antigen-specific tumor-infiltrating T cells. Nature Communications. 2021;12(1):1047. <https://doi.org/10.1038/s41467-021-21383-1>.

46. Bauman JE, Ohr J, Gooding WE, Ferris RL, Duvvuri U, Kim S, et al. Phase I Study of Ficlatuzumab and Cetuximab in Cetuximab-Resistant, Recurrent/Metastatic Head and Neck Cancer. Cancers. 2020;12(6):1537. <https://doi.org/10.3390/cancers12061537>.

47. Bauman JE, Duvvuri U, Ferris RL, Ohr J, Gooding WE, Kim S, et al. Phase I study of the anti-HGF monoclonal antibody (mAb), ficlatuzumab, and cetuximab in cetuximab-resistant, recurrent/metastatic (R/M) head and neck squamous cell carcinoma (HNSCC). Journal of Clinical Oncology. 2017;35(15_suppl):6038-. <https://doi.org/10.1200/JCO.2017.35.15_suppl.6038>.

48. Dunn LA, Riaz N, Fury MG, McBride SM, Michel L, Lee NY, et al. A Phase 1b Study of Cetuximab and BYL719 (Alpelisib) Concurrent with Intensity Modulated Radiation Therapy in Stage III-IVB Head and Neck Squamous Cell Carcinoma. International journal of radiation oncology, biology, physics. 2020;106(3):564-70. <https://doi.org/10.1016/j.ijrobp.2019.09.050>.

49. Karam SD, Reddy K, Blatchford PJ, Waxweiler T, DeLouize AM, Oweida A, et al. Final Report of a Phase I Trial of Olaparib with Cetuximab and Radiation for Heavy Smoker Patients with Locally Advanced Head and Neck Cancer. Clinical cancer research : an official journal of the American Association for Cancer Research. 2018;24(20):4949-59. <https://doi.org/10.1158/1078-0432.ccr-18-0467>.

50. Siu LL, Even C, Mesía R, Remenar E, Daste A, Delord JP, et al. Safety and Efficacy of Durvalumab With or Without Tremelimumab in Patients With PD-L1-Low/Negative Recurrent or Metastatic HNSCC: The Phase 2 CONDOR Randomized Clinical Trial. JAMA oncology. 2019;5(2):195-203. <https://doi.org/10.1001/jamaoncol.2018.4628>.

51. Dillon MT, Grove L, Newbold KL, Shaw H, Brown NF, Mendell J, et al. Patritumab with Cetuximab plus Platinum-Containing Therapy in Recurrent or Metastatic Squamous Cell Carcinoma of the Head and Neck: An Open-Label, Phase Ib Study. Clinical cancer research : an official journal of the American Association for Cancer Research. 2019;25(2):487-95. <https://doi.org/10.1158/1078-0432.ccr-18-1539>.

52. Burtness B, Harrington KJ, Greil R, Soulières D, Tahara M, de Castro G, Jr., et al. Pembrolizumab alone or with chemotherapy versus cetuximab with chemotherapy for recurrent or metastatic squamous cell carcinoma of the head and neck (KEYNOTE-048): a randomised, open-label, phase 3 study. Lancet (London, England). 2019;394(10212):1915-28. <https://doi.org/10.1016/s0140-6736(19)32591-7>.

53. Ferris RL, Haddad R, Even C, Tahara M, Dvorkin M, Ciuleanu TE, et al. Durvalumab with or without tremelimumab in patients with recurrent or metastatic head and neck squamous cell carcinoma: EAGLE, a randomized, open-label phase III study. Annals of oncology : official journal of the European Society for Medical Oncology. 2020;31(7):942-50. <https://doi.org/10.1016/j.annonc.2020.04.001>.

54. Anderson CM, Lee CM, Saunders DP, Curtis A, Dunlap N, Nangia C, et al. Phase IIb, Randomized, Double-Blind Trial of GC4419 Versus Placebo to Reduce Severe Oral Mucositis Due to Concurrent Radiotherapy and Cisplatin For Head and Neck Cancer. J Clin Oncol. 2019;37(34):3256-65. <https://doi.org/10.1200/jco.19.01507>.

55. Day D, Prawira A, Spreafico A, Waldron J, Karithanam R, Giuliani M, et al. Phase I trial of alpelisib in combination with concurrent cisplatin-based chemoradiotherapy in patients with locoregionally advanced squamous cell carcinoma of the head and neck. Oral Oncol. 2020;108:104753. <https://doi.org/10.1016/j.oraloncology.2020.104753>.

56. Rodriguez CP, Wu Q, Voutsinas J, Fromm JR, Jiang X, Pillarisetty VG, et al. A Phase II Trial of Pembrolizumab and Vorinostat in Recurrent Metastatic Head and Neck Squamous Cell Carcinomas and Salivary Gland Cancer. Clinical Cancer Research. 2020;26(4):837. <https://doi.org/10.1158/1078-0432.CCR-19-2214>.

57. Plaschke CC, Johannesen HH, Hansen RH, Hendel HW, Kiss K, Gehl J, et al. The DAHANCA 32 study: Electrochemotherapy for recurrent mucosal head and neck cancer. Head & neck. 2019;41(2):329-39. <https://doi.org/10.1002/hed.25454>.

58. Oppelt P, Ley J, Daly M, Rich J, Paniello R, Jackson RS, et al. nab-Paclitaxel and cisplatin followed by cisplatin and radiation (Arm 1) and nab-paclitaxel followed by cetuximab and radiation (Arm 2) for locally advanced head and neck squamous-cell carcinoma: a multicenter, non-randomized phase 2 trial. Medical oncology (Northwood, London, England). 2021;38(4):35. <https://doi.org/10.1007/s12032-021-01479-w>.

59. Powell SF, Gold KA, Gitau MM, Sumey CJ, Lohr MM, McGraw SC, et al. Safety and Efficacy of Pembrolizumab With Chemoradiotherapy in Locally Advanced Head and Neck Squamous Cell Carcinoma: A Phase IB Study. J Clin Oncol. 2020;38(21):2427-37. <https://doi.org/10.1200/jco.19.03156>.

60. Weiss J, Sheth S, Deal AM, Grilley Olson JE, Patel S, Hackman TG, et al. Concurrent Definitive Immunoradiotherapy for Patients with Stage III–IV Head and Neck Cancer and Cisplatin Contraindication. Clinical Cancer Research. 2020;26(16):4260. <https://doi.org/10.1158/1078-0432.CCR-20-0230>.

61. Harrington KJ, Kong A, Mach N, Chesney JA, Fernandez BC, Rischin D, et al. Talimogene Laherparepvec and Pembrolizumab in Recurrent or Metastatic Squamous Cell Carcinoma of the Head and Neck (MASTERKEY-232): A Multicenter, Phase 1b Study. Clinical cancer research : an official journal of the American Association for Cancer Research. 2020;26(19):5153-61. <https://doi.org/10.1158/1078-0432.ccr-20-1170>.

62. Siano M, Molinari F, Martin V, Mach N, Früh M, Freguia S, et al. Multicenter Phase II Study of Panitumumab in Platinum Pretreated, Advanced Head and Neck Squamous Cell Cancer. The oncologist. 2017;22(7):782-e70. <https://doi.org/10.1634/theoncologist.2017-0069>.

63. Sun XS, Sire C, Tao Y, Martin L, Alfonsi M, Prevost JB, et al. A phase II randomized trial of pembrolizumab versus cetuximab, concomitant with radiotherapy (RT) in locally advanced (LA) squamous cell carcinoma of the head and neck (SCCHN): First results of the GORTEC 2015-01 “PembroRad” trial. Journal of Clinical Oncology. 2018;36(15_suppl):6018-. <https://doi.org/10.1200/JCO.2018.36.15_suppl.6018>.

64. Bourhis J, Sire C, Tao Y, Martin L, Alfonsi M, Prevost JB, et al. LBA38 Pembrolizumab versus cetuximab, concomitant with radiotherapy (RT) in locally advanced head and neck squamous cell carcinoma (LA-HNSCC): Results of the GORTEC 2015-01 &#x201c;PembroRad&#x201d; randomized trial. Annals of Oncology. 2020;31:S1168. <https://doi.org/10.1016/j.annonc.2020.08.2268>.

65. Fuereder T, Minichsdorfer C, Mittlboeck M, Wagner C, Oberndorfer F, Müllauer L, et al. 921P Pembrolizumab plus docetaxel for the treatment of recurrent metastatic head and neck cancer: A prospective phase I/II study. Annals of Oncology. 2020;31:S665. <https://doi.org/10.1016/j.annonc.2020.08.1036>.

66. Gillison ML, Ferris RL, Harris J, Colevas AD, Mell LK, Kong C, et al. Safety and disease control achieved with the addition of nivolumab (Nivo) to chemoradiotherapy (CRT) for intermediate (IR) and high-risk (HR) local-regionally advanced head and neck squamous cell carcinoma (HNSCC): RTOG Foundation 3504. Journal of Clinical Oncology. 2019;37(15_suppl):6073-. <https://doi.org/10.1200/JCO.2019.37.15_suppl.6073>.

67. Elbers JBW, Al-Mamgani A, Tesseslaar MET, van den Brekel MWM, Lange CAH, van der Wal JE, et al. Immuno-radiotherapy with cetuximab and avelumab for advanced stage head and neck squamous cell carcinoma: Results from a phase-I trial. Radiotherapy and oncology : journal of the European Society for Therapeutic Radiology and Oncology. 2020;142:79-84. <https://doi.org/10.1016/j.radonc.2019.08.007>.

68. Tao Y, Aupérin A, Sun X, Sire C, Martin L, Coutte A, et al. Avelumab-cetuximab-radiotherapy versus standards of care in locally advanced squamous-cell carcinoma of the head and neck: The safety phase of a randomised phase III trial GORTEC 2017-01 (REACH). European journal of cancer (Oxford, England : 1990). 2020;141:21-9. <https://doi.org/10.1016/j.ejca.2020.09.008>.

69. Zuur L, Vos JL, Elbers JB, Krijgsman O, Qiao X, van der Leun A, et al. LBA40 Neoadjuvant nivolumab and nivolumab plus ipilimumab induce (near-) complete responses in patients with head and neck squamous cell carcinoma: The IMCISION trial. Annals of Oncology. 2020;31:S1169. <https://doi.org/10.1016/j.annonc.2020.08.2270>.

70. Chung CH, Bonomi M, Steuer CE, Li J, Bhateja P, Johnson M, et al. Concurrent Cetuximab and Nivolumab as a Second-Line or beyond Treatment of Patients with Recurrent and/or Metastatic Head and Neck Squamous Cell Carcinoma: Results of Phase I/II Study. Cancers. 2021;13(5):1180. <https://doi.org/10.3390/cancers13051180>.

71. Hsieh C-Y, Lein M-Y, Yang S-N, Wang Y-C, Lin Y-J, Lin C-Y, et al. Dose-dense TPF induction chemotherapy for locally advanced head and neck cancer: a phase II study. BMC Cancer. 2020;20(1):832. <https://doi.org/10.1186/s12885-020-07347-6>.

72. Bossi P, Bergamini C, Miceli R, Cova A, Orlandi E, Resteghini C, et al. Salivary Cytokine Levels and Oral Mucositis in Head and Neck Cancer Patients Treated With Chemotherapy and Radiation Therapy. International journal of radiation oncology, biology, physics. 2016;96(5):959-66. <https://doi.org/10.1016/j.ijrobp.2016.08.047>.

73. Bossi P, Di Pede P, Guglielmo M, Granata R, Alfieri S, Iacovelli NA, et al. Prevalence of Fatigue in Head and Neck Cancer Survivors. The Annals of otology, rhinology, and laryngology. 2019;128(5):413-9. <https://doi.org/10.1177/0003489419826138>.

74. Botticelli A, Mezi S, Pomati G, Sciattella P, Cerbelli B, Roberto M, et al. The Impact of Locoregional Treatment on Response to Nivolumab in Advanced Platinum Refractory Head and Neck Cancer: The Need Trial. Vaccines. 2020;8(2):191. <https://doi.org/10.3390/vaccines8020191>.

75. Byrne K, Hallworth P, Monfared AAT, Moshyk A, Shaw JW. Real-World Systemic Therapy Treatment Patterns for Squamous Cell Carcinoma of the Head and Neck in Canada. Current Oncology. 2019;26(2):167-74. <https://doi.org/10.3747/co.26.3946>.

76. Castelli J, Depeursinge A, Devillers A, Campillo-Gimenez B, Dicente Y, Prior JO, et al. PET-based prognostic survival model after radiotherapy for head and neck cancer. European journal of nuclear medicine and molecular imaging. 2019;46(3):638-49. <https://doi.org/10.1007/s00259-018-4134-9>.

77. de Ridder M, de Veij Mestdagh PD, Elbers JBW, Navran A, Zuur CL, Smeele LE, et al. Disease course after the first recurrence of head and neck squamous cell carcinoma following (chemo)radiation. European archives of oto-rhino-laryngology : official journal of the European Federation of Oto-Rhino-Laryngological Societies (EUFOS) : affiliated with the German Society for Oto-Rhino-Laryngology - Head and Neck Surgery. 2020;277(1):261-8. <https://doi.org/10.1007/s00405-019-05676-2>.

78. Galot R, van Marcke C, Helaers R, Mendola A, Goebbels RM, Caignet X, et al. Liquid biopsy for mutational profiling of locoregional recurrent and/or metastatic head and neck squamous cell carcinoma. Oral Oncol. 2020;104:104631. <https://doi.org/10.1016/j.oraloncology.2020.104631>.

79. Grünwald V, Chirovsky D, Cheung WY, Bertolini F, Ahn MJ, Yang MH, et al. Global treatment patterns and outcomes among patients with recurrent and/or metastatic head and neck squamous cell carcinoma: Results of the GLANCE H&N study. Oral Oncol. 2020;102:104526. <https://doi.org/10.1016/j.oraloncology.2019.104526>.

80. Hilke FJ, Muyas F, Admard J, Kootz B, Nann D, Welz S, et al. Dynamics of cell-free tumour DNA correlate with treatment response of head and neck cancer patients receiving radiochemotherapy. Radiotherapy and oncology : journal of the European Society for Therapeutic Radiology and Oncology. 2020;151:182-9. <https://doi.org/10.1016/j.radonc.2020.07.027>.

81. Kim H, Kwon M, Kim B, Jung HA, Sun J-M, Lee S-H, et al. Clinical outcomes of immune checkpoint inhibitors for patients with recurrent or metastatic head and neck cancer: real-world data in Korea. BMC Cancer. 2020;20(1):727. <https://doi.org/10.1186/s12885-020-07214-4>.

82. Martens RM, Noij DP, Koopman T, Zwezerijnen B, Heymans M, de Jong MC, et al. Predictive value of quantitative diffusion-weighted imaging and 18-F-FDG-PET in head and neck squamous cell carcinoma treated by (chemo)radiotherapy. European journal of radiology. 2019;113:39-50. <https://doi.org/10.1016/j.ejrad.2019.01.031>.

83. Martens RM, Koopman T, Noij DP, Pfaehler E, Übelhör C, Sharma S, et al. Predictive value of quantitative (18)F-FDG-PET radiomics analysis in patients with head and neck squamous cell carcinoma. EJNMMI research. 2020;10(1):102. <https://doi.org/10.1186/s13550-020-00686-2>.

84. Nadler E, Joo S, Boyd M, Black-Shinn J, Chirovsky D. Treatment patterns and outcomes among patients with recurrent/metastatic squamous cell carcinoma of the head and neck. Future oncology (London, England). 2019;15(7):739-51. <https://doi.org/10.2217/fon-2018-0572>.

85. Noij DP, Martens RM, Koopman T, Hoekstra OS, Comans EFI, Zwezerijnen B, et al. Use of Diffusion-Weighted Imaging and (18)F-Fluorodeoxyglucose Positron Emission Tomography Combined With Computed Tomography in the Response Assessment for (Chemo)radiotherapy in Head and Neck Squamous Cell Carcinoma. Clinical oncology (Royal College of Radiologists (Great Britain)). 2018;30(12):780-92. <https://doi.org/10.1016/j.clon.2018.09.007>.

86. Pitak-Arnnop P, Witohendro LK, Meningaud JP, Subbalekha K, Iamaroon A, Sirintawat N, et al. Which characteristics can be expected from p16(+)-squamous cell carcinomas of the posterior oral cavity and oropharynx? - Distinctive results from Central Germany. Journal of stomatology, oral and maxillofacial surgery. 2020;121(3):213-8. <https://doi.org/10.1016/j.jormas.2019.10.013>.

87. Porter A, Natsuhara M, Daniels GA, Patel SP, Sacco AG, Bykowski J, et al. Next generation sequencing of cell free circulating tumor DNA in blood samples of recurrent and metastatic head and neck cancer patients. Translational Cancer Research. 2020;9(1):203-9. <https://doi.org/10.21037/tcr.2019.12.70>.

88. Smirk R, Kyzas P. Outcome of salvage procedures for recurrent oral and oropharyngeal cancer. The British journal of oral & maxillofacial surgery. 2018;56(9):847-53. <https://doi.org/10.1016/j.bjoms.2018.09.008>.

89. Sridharan V, Rahman RM, Huang RY, Chau NG, Lorch JH, Uppaluri R, et al. Radiologic predictors of immune checkpoint inhibitor response in advanced head and neck squamous cell carcinoma. Oral Oncol. 2018;85:29-34. <https://doi.org/10.1016/j.oraloncology.2018.08.005>.

90. Velez MA, Wang PC, Hsu S, Chin R, Beron P, Abemayor E, et al. Prognostic significance of HPV status in the re-irradiation of recurrent and second primary cancers of the head and neck. American journal of otolaryngology. 2018;39(3):257-60. <https://doi.org/10.1016/j.amjoto.2018.01.011>.
